# Incidence and evolutionary relevance of autotriploid cytotypes in a relict member of the genus *Daphne* (Thymelaeaceae)

**DOI:** 10.1093/aobpla/plad056

**Published:** 2023-08-30

**Authors:** Zuzana Gajdošová, Marek Svitok, Veronika Cetlová, Lenka Mártonfiová, Jaromír Kučera, Vladislav Kolarčik, Bogdan-Iuliu Hurdu, Ioana-Minodora Sîrbu, Ingrid Turisová, Peter Turis, Marek Slovák

**Affiliations:** Department of Evolution and Systematics, Institute of Botany, Plant Sciences and Biodiversity Centre, Slovak Academy of Sciences, Dúbravská cesta 9, SK-845 23 Bratislava, Slovak Republic; Department of Biology and General Ecology, Faculty of Ecology and Environmental Sciences, Technical University in Zvolen, Ul. T. G. Masaryka 24, SK-960 01 Zvolen, Slovak Republic; Department of Forest Ecology, Czech University of Life Sciences Prague, CZ-16 521 Suchdol, Praha 6, Czech Republic; Department of Evolution and Systematics, Institute of Botany, Plant Sciences and Biodiversity Centre, Slovak Academy of Sciences, Dúbravská cesta 9, SK-845 23 Bratislava, Slovak Republic; Botanical Garden of Pavol Jozef Šafárik University in Košice, Mánesova 23, SK-043 52 Košice, Slovak Republic; Department of Evolution and Systematics, Institute of Botany, Plant Sciences and Biodiversity Centre, Slovak Academy of Sciences, Dúbravská cesta 9, SK-845 23 Bratislava, Slovak Republic; Institute of Biology and Ecology, Faculty of Science, Pavol Jozef Šafárik University in Košice, Mánesova 23, SK-041 54 Košice, Slovak Republic; Department of Taxonomy and Evolution, Institute of Biological Research, 48 Republicii St., R-400015 Cluj-Napoca, Romania; Faculty of Biology, University of Bucharest, Splaiul Independenței 91–95, R-050095Bucharest, Romania; Department of Biology and Ecology, Faculty of Natural Sciences, Matej Bel University in Banská Bystrica, Tajovského 40, SK-974 01 Banská Bystrica, Slovak Republic; Department of Biology and Ecology, Faculty of Natural Sciences, Matej Bel University in Banská Bystrica, Tajovského 40, SK-974 01 Banská Bystrica, Slovak Republic; Department of Evolution and Systematics, Institute of Botany, Plant Sciences and Biodiversity Centre, Slovak Academy of Sciences, Dúbravská cesta 9, SK-845 23 Bratislava, Slovak Republic; Department of Botany, Faculty of Science, Charles University, Benátská 2, CZ-128 01 Praha, Czech Republic

**Keywords:** Carpathians, *Daphne*, endemics, genome size stasis, ITS, Pannonian Basin, pollen fertility, polyploidy, relicts, triploids

## Abstract

Odd ploidy-level cytotypes in sexually reproducing species are considered a dead end due to absent or reduced fertility. If sterility is only partial, however, their contribution to the population gene pool can be augmented by longevity and clonal growth. To test this, we investigated the cytotype origin and spatial pattern, and pollen viability in three relict shrub species of the genus *Daphne* (Thymelaeaceae Juss.) in central Europe. *Daphne cneorum* subsp. *cneorum* is a widespread European species that has a broad ecological amplitude, whereas *D*. *cneorum* subsp. *arbusculoides* and *D. arbuscula* are narrow endemics of the western Pannonian Plain and the Western Carpathians, respectively. Our study confirmed that all three taxa are diploid. However, of more than a thousand analysed individuals of *D. cneorum* subsp*. cneorum*, five in four different populations were triploid. Our data indicate that these triploids most likely originate from recurrent autopolyploidization events caused by the fusion of reduced and unreduced gametes. High pollen viability was observed in all three taxa and in both diploid and triploid cytotypes, ranging from 65 to 100 %. Our study highlights the significant role of odd ploidy-level cytotypes in interploidy gene flow, calling for more research into their reproduction, genetic variability, and overall fitness. Interestingly, while the endemic *D. arbuscula* differs from *D. cneorum* based on genetic and genome size data, *D. cneorum* subsp. *arbusculoides* was indistinguishable from *D. cneorum* subsp. *cneorum*. However, our study reveals that the subspecies differ in the number of flowers per inflorescence. This is the first comprehensive cytogeographic study of this intriguing genus at a regional scale, and in spite of its karyological stability, it contributes to our understanding of genomic evolution in plant species with a wide ecological amplitude.

## Teaser

The presented study sheds light on the emergence of odd-ploidy cytotypes in diploid species, specifically in long-lived relic shrubs from the genus *Daphne* (Thymeleaceae). Rare odd-ploidy cytotypes are generally considered maladaptive in sexual diploid species due to reduced fitness and fertility. However, our research revealed the independent and recurring evolution of triploid cytotypes, most likely arising via the fusion of reduced and unreduced gametes in exclusively diploid systems and exhibiting significant pollen fertility. Their even partial pollen fertility, especially, in the context of their longevity, can contribute to a more diverse gene pool in their parental diploid populations or even to the spontaneous emergence of new cytotypes. Additionally, we demonstrated that although *Daphne cneorum* exhibits a large ecological amplitude, spanning various bedrock substrates and altitudinal ranges, its diploid genomes remain highly stable with no ecologically driven genome size expansions or reductions, as previously observed in many species with large ecological amplitudes.

## Introduction

Quaternary climate change, coupled with human influence in the Anthropocene, has had a crucial effect on the evolution of biomes and the present-day distribution patterns of all organisms (Hewitt 1996, 2000; Lewis and Maslin 2015). Extensive environmental disturbances have induced species range shifts and habitat fragmentation, posing a substantial threat to the survival of plant populations and natural communities. Numerous plant species could only withstand such significant environmental perturbations in isolated, albeit ecologically stable habitat islands in areas that are inaccessible to humans and livestock (Larson *et al.* 2000; Lavergne *et al.* 2005; Huang *et al.* 2015; Tang *et al.* 2018). The complex evolutionary histories of these relict populations may augment their evolutionary dynamics, resulting in genomic rearrangements, genome size diversification through the accumulation of transposable elements and whole-genome duplications (Soltis *et al.* 2003; Macas *et al.* 2015; Schubert and Vu 2016; Van de Peer *et al.* 2017). Genome size variation is often evidenced in widespread and ecologically tolerant species, with structural genome rearrangements driven by environmental heterogeneity (Šmarda and Bureš 2006; Slovák *et al.* 2009; Nunvářová Kabátová *et al.* 2019). Polyploid cytotypes are also common in relict species (e.g. Siljak-Yakovlev *et al.* 2008; Besnard and Baali-Cherif 2009; García-Verdugo *et al.* 2013). Morphological, anatomical and physiological changes associated with polyploidization may lead to improved fitness and a shift in ecological requirements in novel cytotypes, enhancing their ability to survive and adapt to environmental changes (Levin 2002; Otto 2007). Odd ploidy cytotypes may emerge either through the fusion of reduced and unreduced gametes within diploid species or through evolution as a result of heteroploid hybridization (Ramsey and Schemske 1998; Husband *et al.* 2013). In certain heteroploid systems, gene flow between different cytotypes can contribute to the establishment of newly formed polyploids (Burton and Husband 2001; Čertner *et al.* 2017; Kolář *et al.* 2017). Occasional triploids are typically considered maladaptive in sexual diploid species due to their decreased fitness and fertility (Ramsey and Schemske 1998; Joly and Bruneau 2004). Nevertheless, their persistence, frequency and potential evolutionary significance in exclusively diploid systems may be remarkably greater in long-lived species such as shrubs, trees and clonal species, and may even exhibit better adaptation to more stressful environments than their diploid progenitors (Mock *et al.* 2012). Consequently, a longer lifespan and at least partial fertility in triploids can contribute to an enriched gene pool in their parental diploid populations (Lefort *et al.* 2000; Blakesley *et al.* 2002; Dzialuk *et al.* 2007; Besnard and Baali-Cherif 2009; Mock *et al.* 2012).

**Figure 1.**
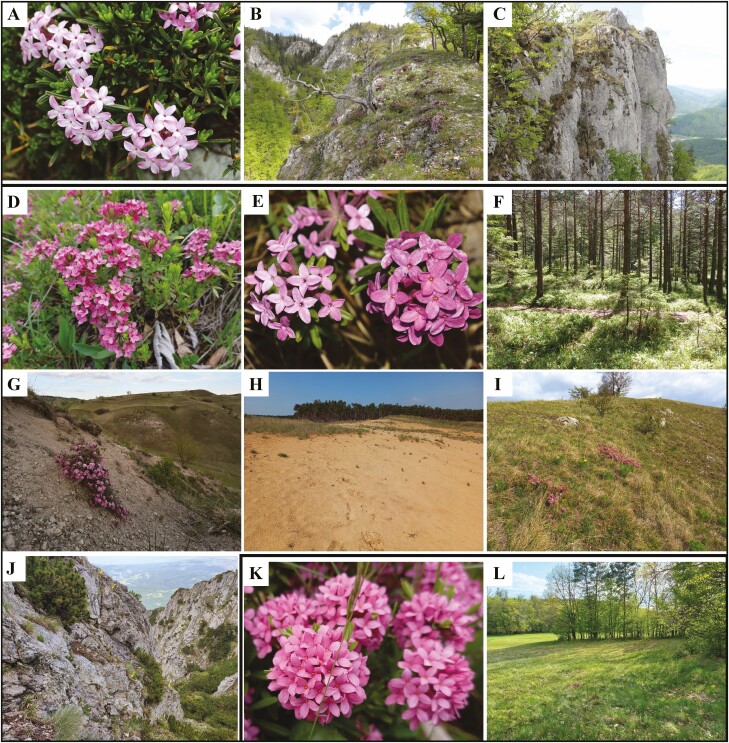
Studied taxa and their habitats: (A) *Daphne arbuscula*; (B and C) calcareous cliffs in Muránska Planina Mts., Western Carpathians (SK-DA 7 and SK-DA 8); (D) *D. cneorum* subsp. *cneorum*—diploid cytotype; Sokol, Veľká Fatra Mts., Western Carpathians (SK-DC 6); (E) *D. cneorum* subsp. *cneorum—*triploid cytotype; Záhorská nížina Lowland, Pannonian Basin (SK-DC 1); (F) calcareous relict pine forest, Karlschutt, Hochschwab Mts., Eastern Alps Mts. (AT-DC 11); (G) steppic grasslands on gypsum, Sfăraș-Jebucu, Transylvanian Plateau, Romania (RO-DC 19); (H) siliceous sands, Záhorská nížina Lowland, Pannonian Basin (SK-DC 1); (I) steppic calcareous grasslands, Gánt, Transdanubian Mts., Pannonian Basin (HU-DC 17); (J) subalpine calcareous rocks, Turnu peak, Piatra Craiului Mts., Southern Carpathians (RO-DC 25); (K) *D. cneorum* subsp. *arbusculoides* (HU-DCA 2); (L) grassland and forest margins on the siliceous substrate, Őrség region, Pannonian Basin (HU-DCA 2). Photos A–D, F, H, I, K, L credits to Z. Gajdošová; E credit to J. Kučera, G credit to B.-I. Hurdu; J credit to A. Indreica.

We investigate the karyological variability of three relict taxa of the genus *Daphne* (Thymelaeaceae Juss.) assumed to have evolved in the Quaternary or even the Tertiary epoch (Browicz and Gostyńska-Jakuszewska 1967; Muller 1997; Halda 2001; Melnyk and Baransky 2020), namely *D. arbuscula*, *D. cneorum* subsp. *cneorum* and *D. cneorum* subsp. *arbusculoides*. All three taxa are evergreen, long-lived small shrubs with strongly fragrant, pinkish, insect-pollinated flowers and fruits (drupes) dispersed by animals. They are reported to be allogamous, lacking noticeable long-distance dispersal systems, and to have reduced seed production and germination, accompanied by a high level of clonal spread (e.g. Erdelská and Turis 1995; Šedivá and Žlebčík 2010; Gajdošová 2020; Di Sacco *et al.* 2021). The phylogenetic relationships and even clear taxonomic identities of *D. arbuscula* and *D. cneorum* subsp. *arbusculoides* have never been clarified using genetic data. *Daphne arbuscula* is a narrow endemic to the Western Carpathians (Muránska Planina Mts.), occurring in mostly small, isolated patches at mid-altitude on relict limestone cliffs (Fig. 1A–C). Despite its limited distribution, *D. arbuscula* is not stenotopic; distinct groups of micro-localities show different plant associations and microclimatic conditions in regard to temperature and humidity (Valachovič and Jarolímek 1994; Erdelská and Turis 1995; Uhlířová and Bernátová 2003, 2004; Kochjarová *et al.* 2004). In contrast, *D. cneorum* is widespread and can be found across southern, central, and eastern Europe. Its island-type distribution has likely been influenced by habitat fragmentation associated with anthropogenic factors (Browicz and Gostyńska-Jakuszewska 1967; Muller 1997; Orsenigo *et al.* 2019; Melnyk and Baransky 2020). The nominate subspecies occurs in a variety of relict habitats, from the lowlands (≥120 m a.s.l.) to the subalpine zone (≤1796 m a.s.l., **see****Supporting Information—Table S1**), also occurring in various bedrock types. It prefers relictual grasslands and oak or pine forests on limestone bedrock, but it can also be found in siliceous grasslands, winded sands, and occasionally also on serpentine or gypsum bedrock localities (Fig. 1D–J; Webb and Ferguson 1968; Halda 1976; Orsenigo *et al.* 2019). In contrast, *D. cneorum* subsp. *arbusculoides* is narrowly distributed in the south-westernmost part of the Pannonian Basin, with micro-localities in SW Hungary, NE Slovenia, and SE Austria (Fig. 1K and L), occurring in grassland-forest habitats on siliceous bedrock. Morphologically, *D. cneorum* subsp. *arbusculoides* differs from the nominate subspecies by having more erect branches and leaves with revolute margins (Tuzson 1911; Soó 1971; Halda 2001).

The vast majority of members of the genus *Daphne*, including *D. arbuscula* and *D. cneorum* subsp. *cneorum*, are diploids with 2*n* = 2*x* = 18, with only a few published instances of polyploidy, including triploids (Goldblatt and Johnson 1979; Rice *et al.* 2015; **see****Supporting Information—Table S2**). No karyological data for *D. cneorum* subsp. *arbusculoides* have been published to date. During our pilot investigation on the karyological diversity of *D. cneorum* subsp. *cneorum* from the Slovak populations, we identified two individuals with relative genome sizes (RGSs) that deviated from the diploid stage, indicating a triploid level. However, it is unclear whether these individuals are true triploids resulting from the fusion of unreduced and reduced gametes or heteroploid hybrids between diploid and possibly tetraploid individuals (Soltis and Soltis 2000; Kolář *et al.* 2017). Although the reported karyological variability of *D. cneorum* subsp. *cneorum* and *D. arbuscula* is limited, their distribution patterns and ecological setup might indicate a niche shift driven by genome size and ploidy-level diversification (Slovák *et al.* 2009; Nunvářová Kabátová *et al.* 2019). This provides an ideal opportunity to investigate ploidy level and genome size variability of three *Daphne* taxa in relation to spatial patterns in the Carpathians, Eastern Alps, and the Pannonian Plain. Furthermore, because triploid individuals may not be completely sterile and thus contribute to population ploidy-level diversification through gene flow, we also investigate the pollen viability of all studied taxa and cytotypes to determine their evolutionary potential with respect to their contribution to parental gene pools. Notably, our study is the first to provide insight into the genetic variability and divergence of *D. arbuscula* and *D. cneorum*.

## Material and Methods

### Plant material and study sites

**Figure 2.**
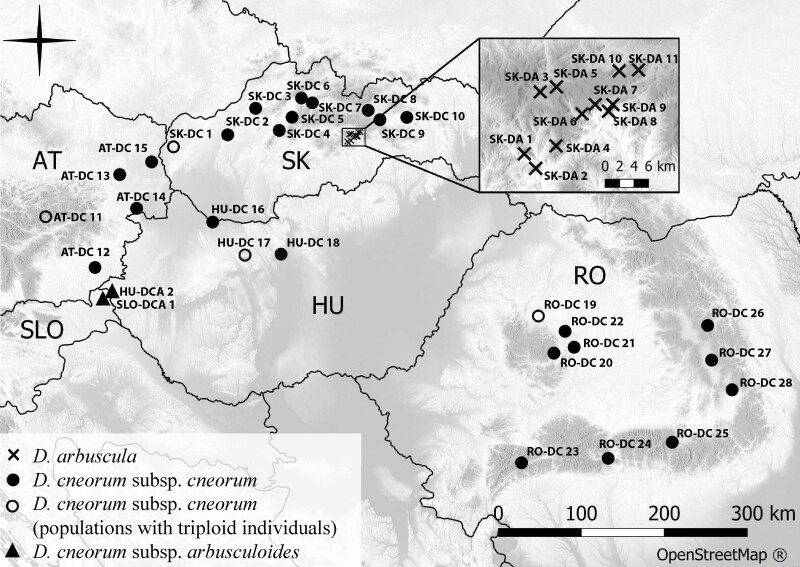
Map of sampled populations from the analysed *Daphne* taxa: DA—*D. arbuscula*; DC—*D. cneorum* subsp. *cneorum*; DCA—*D. cneorum* subsp. *arbusculoides*. Country codes: AT—Austria, HU—Hungary, RO—Romania, SK—Slovakia, SLO—Slovenia. Taxa and cytotypes are indicated by different symbols. Population codes follow **Supporting Information—Table S1**.

We collected material from the entire distribution range of both endemic taxa, namely *D. arbuscula* from the Western Carpathians (11 populations) and *D. cneorum* subsp. *arbusculoides* from the SW Pannonian Basin (two populations) (Fig. 2; **see****Supporting Information—Table S1**). *Daphne cneorum* subsp. *cneorum*, a pan-European species distributed from Spain in the west to Ukraine in the east (Halda 2001), was sampled only in the central and eastern parts of its distribution range, including the Carpathians, the Pannonian Basin, and, to a lesser extent, the NE Alps. However, the 28 sampled populations cover the entire habitat and ecological range reported for this taxon (Fig. 2; **see****Supporting Information—Table S1**; Webb and Ferguson 1968; Halda 1976; Orsenigo *et al.* 2019). For the purpose of our analyses, we used both exsiccated and fresh material (flowers or flower buds, young leaves, and root samples). The sampling effort aimed to cover the entire genetic diversity across each population while avoiding clonality effects (maximizing distance between individuals), reducing any negative impact on the population, and mitigating any damage to individual shrubs by collecting only the number of floral buds necessary for analyses per individual.

### Ploidy level, and relative and absolute genome size estimation

To evaluate ploidy level and estimate RGS, we analysed 5–30(−250) individuals per population, depending on population size (89 individuals in *D. arbuscula*, 1149 diploid and 5 triploid individuals in *Daphne cneorum* subsp. *cneorum*, and 177 individuals in *D. cneorum* subsp. *arbusculoides*; **see****Supporting Information—Table S1**). Absolute genome size (AGS) was estimated using at least three individuals per population from selected populations (9 individuals in *D. arbuscula*, 28 diploid individuals, and 1 triploid individual in *Daphne cneorum* subsp. *cneorum*, and 10 individuals in *D. cneorum* subsp. *arbusculoides*; **see****Supporting Information—Table S1**). Both measurements were performed using Partec CyFlow ML (Partec GmbH, Münster, Germany) equipped with a green solid-state laser (Cobolt Samba 532 nm, 150 mW; Cobolt, Stockholm, Sweden) as the excitation source for AGS or a UV lamp or HBO 100 W mercury arc lamp for RGS. Samples were prepared according to the standard protocol using a general-purpose buffer and propidium iodide (PI) for AGS (Loureiro *et al.* 2007), or the two-step procedure with Otto buffers and 4ʹ,6-diamidino-2-phenylindole (DAPI) for RGS (Otto 1990; Doležel *et al.* 2007). *Bellis perennis* was chosen as an internal standard (2*C* DNA = 3.38 pg; J. Suda unpubl. in: Schönswetter *et al.* 2007). For RGS, three to five samples were simultaneously evaluated in larger populations. If samples showed traces of RGS variation, we aimed to detect the real genome size difference using simultaneous analyses. At least 5000 nuclei for AGS and 3000 nuclei for RGS were measured, with the coefficients of variation of the G0/G1 peaks for both samples and the internal standard never exceeding 5 %. For AGS, at least three independent measurements were performed on consecutive days for each individual, with the inter-day variation threshold of the three iterations per sample being set at 3 % (Greilhuber and Obermayer 1997). If this variance was greater than 3 %, the outlying value was discarded and the corresponding sample was reanalysed. The AGS (2*C* value) estimation was computed as the ratio of the sample G0/G1 peak position to the standard G0/G1 peak position multiplied by the 2*C* DNA content of the standard (pg DNA; Doležel and Bartoš 2005). RGS was calculated as the ratio between the positions of the sample and standard G0/G1 peaks. Flow cytometric histograms (both PI and DAPI) were assessed using FloMax version 2.70 software (Partec Gmbh, Münster, Germany). The GC content was calculated separately for each pair of RGS and AGS measurements using the protocol by Šmarda *et al.* (2008) and subsequently averaged for each taxon and cytotype.

### Chromosome counting

Somatic chromosome numbers (2*n*) were counted in fresh roots or very young flower bud meristems obtained from wild plants or rooting shoots of *D. arbuscula*, diploid and triploid cytotypes of *D. cneorum* subsp. *cneorum*, and *D. cneorum* subsp. *arbusculoides.* Individuals with counted chromosome numbers were simultaneously analysed for AGS and RGS and served as references for estimating ploidy in the remaining cytotypes and taxa. Freshly collected meristems were pre-treated in a 0.002 M water solution of 8-hydroxyquinoline for 3–5 h at low temperature (4 °C), washed in distilled water, fixed in a 3:1 mixture of 96 % ethanol and 98 % acetic acid for 24 h, and stored in 75 % ethanol. Samples were washed for 10 min in distilled water before and after being macerated in a 1:1 mixture of 35 % HCl and 96 % ethanol for 3 min prior to analyses. Microscopic slides and squashes were prepared using the cellophane square technique (Murín 1960). The slides were stained with a 7 % Giemsa stain solution, dried, and observed microscopically in a drop of immersion oil.

All chromosome spreads were analysed under 1000-fold magnification using a light microscope, and micrographs were taken using a ZEISS Axiocam 105 colour (Carl Zeiss, Vienna, Austria) and AxioVision LE64 v. 4.9.1.0 (Carl Zeiss, Microscopy GmbH).

### Pollen viability and flower number

Fresh flower buds were dried and stored in silica gel. Pollen viability was analysed using a modified Alexander’s stain, following the standard protocol (Peterson *et al.* 2010). Viable and non-viable pollen grains were discriminated based on shape, size, and colour. Specifically, magenta–red pollen grains of regular shape were considered viable, while bluish–green, greenish–purple, and shrunken grains were considered non-viable (**see****Supporting Information—Fig. S1**; Peterson *et al.* 2010). A light microscope was used to examine pollen grains at 400-fold magnification, and micrographs were taken using a ZEISS Axiocam 105 colour (Carl Zeiss, Vienna, Austria) and AxioVision LE64 v. 4.9.1.0 (Carl Zeiss, Microscopy GmbH). Pollen viability was calculated as the percentage of non-aborted pollen grains from at least 100 analysed grains per flower, individualizing the standard deviation. Where possible, we examined five individuals per population with one flower per individual (18 individuals in *D. arbuscula*, 52 diploid and 3 triploid individuals in *Daphne cneorum* subsp. *cneorum*, and 10 individuals in *D. cneorum* subsp. *arbusculoides*; **see****Supporting Information—Table S1**).

In addition, to provide further evidence about the potential shifts in fitness and fertility of triploids, we tested for ploidy-induced shifts in flower number per inflorescence between cytotypes and taxa. Flowers were counted in one inflorescence per individual; usually from 5 to 15 individuals per selected population, or exceptionally more (40 individuals in *D. arbuscula*, 158 diploid and 3 triploid individuals in *Daphne cneorum* subsp. *cneorum*, and 95 individuals in *D. cneorum* subsp. *arbusculoides*; **see****Supporting Information—Table S1**). Only three out of five triploid individuals were examined for pollen viability and number of flowers, as the other two lacked flower buds and/or complete inflorescences at the time of collection. In triploids, pollen viability was determined using four buds per individual, whereas flower number was evaluated using 1–3 inflorescences per plant.

## Molecular Analysis

### DNA extraction, molecular markers and PCR amplification

Total genomic DNA was extracted from silica gel-conserved, young, intact leaves using a GeneAll® ExgeneTM Plant SV mini kit (GeneAll Biotechnology, Songpa-gu, Seoul, Korea), following the manufacturer’s protocol, with minor modifications (added PVP and 6 µl of RNase A). Genetic variability was tested using the ITS1-5.8S-ITS2 region (ITS) of nuclear ribosomal DNA (nrDNA; 26 individuals, 3 taxa) and the *ndhF-rpl32* region of chloroplast DNA (cpDNA; 23 individuals, 3 taxa). The ITS region was amplified using primers ITS4 and ITS5, and internal primers ITS2 and ITS3, when necessary (White *et al.* 1990). The *ndhF-rpl32* region was amplified using ndhF and rpl32-R primers (Shaw *et al.* 2007). Polymerase chain reaction (PCR) amplifications were performed in a total volume of 10 µl containing 1 µl of template DNA, 0.25 U DreamTaq DNA polymerase (Thermo Fisher Scientific Inc., Waltham, MA, USA), 1 µl of 10× DreamTaq buffer, 0.2 µl of dNTP Mix (10 mM each), and 0.2 µl of 10 mM each forward and reverse primers. The following PCR conditions were used: 95 °C for 3 min, 30 cycles (95 °C for 30 s, 51 °C for 30 s, 72 °C for 1 min), and 72 °C for 10 min for the ITS amplification; 80 °C for 5 min, 30 cycles (95 °C for 1 min, 52 °C for 1 min, followed by a 5 % ramp to 65 °C, 65 °C for 4 min), and 65 °C for 5 min for the *ndhF-rpl32* region. PCR products were purified using a mixture (1:2) of Exonuclease I and FastAP Thermosensitive Alkaline Phosphatase (Thermo Fisher Scientific Inc.) or using NucleoSpin® Gel and PCR Clean-up (Macherey-Nagel GmbH & Co. KG, Germany) and sequenced by the ABI 3730xl DNA analyser at Eurofins Genomics Company (Konstanz, Germany).

### Sequence processing and alignment

Sequences were edited and aligned using Geneious software R7.1.9 (Biomatters Ltd., Auckland, New Zealand). Both the ITS and *ndhF-rpl32* datasets were supplemented with sequences of other related *Daphne* species and a selection of Thymelaeaceae outgroups available in GenBank. Between- and within-species variability was analysed using Bayesian analyses as conducted in MrBayes v.3.2.7a (Ronquist and Huelsenbeck 2003; Ronquist *et al.* 2012) and randomized accelerated maximum likelihood (ML, RAxML) algorithm (Stamatakis 2014). Prior to carrying out phylogenetic inferences, the best-fit models of nucleotide substitutions were assessed independently for each partition of the nucleotide data partition in jModelTest v.2.1.10 (Darriba *et al.* 2012) using the Akaike information criterion (AIC; Akaike 1974). The nrDNA dataset included three partitions and nucleotide substitution models: (i) non-coding ITS1 and ITS2 (HKY + I); (ii) coding 5.8S rDNA (SYM + I); (iii) indels within the ITS dataset. The *ndhF-rpl32* plastid region had a single partition and the TVM + G model. Indels present in the sequence alignments were coded as binary characters using FastGap 1.2 software (Borchsenius 2009) according to the simple indel coding approach (Simmons and Ochoterena 2000). Bayesian analyses (BI) were conducted with four Markov chain Monte Carlo (MCMC) and two independent runs for 10–15 million generations, with a sampling frequency of every 100th generation. The first 10 % of trees were discarded as ‘burn-in’. RAxML analyses were run using rapid bootstrapping for 1000 replicates. Analyses were run on the CIPRES Science Gateway (Miller *et al.* 2010). Phylogenetic trees were visualized, and bootstrap support (BS) and posterior probabilities (PP) values were appended to trees using FigTree (v1.4.4). Bootstrap support was categorized according to the following criteria: strong (>85 %), moderate (70–85 %), weak (50–69 %) and poor (<50 %). Posterior probability values of 0.90 and above were considered significant, and those values below 0.90 were regarded as non-significant.

### Data analysis

Inter-specific and ploidy-level variability in RGS, AGS, pollen viability, and the number of flowers among the three *Daphne* taxa were assessed by generalized linear mixed models (GLMMs; Bolker *et al.* 2009). Differences in non-integer values of RGS and AGS were tested using GLMMs with a Gaussian distribution and identity link function. A Poisson model with a logarithmic link was used to compare differences in flower counts. Finally, a binomial GLMM with a logit link was employed to evaluate pollen viability, that is, the proportion of viable pollen grains out of the total grains examined. Since our sampling design involved measurements of several plants at each site, we treated the population as a random intercept in the models to deal with a potential autocorrelation within sites. An additional random effect was included in the binomial GLMM to account for multiple pollen samples from the same plants nested within populations. The performance of each model was evaluated using a simulation-based approach to residual diagnostic (Dunn and Smyth 1996); no major deviations from the assumptions behind the models were observed. The statistical significance of the Gaussian GLMMs was evaluated using *F*-tests with Kenward-Roger adjusted degrees of freedom (Kenward and Roger 1997). The significance of the Poisson and binomial GLMM was assessed using likelihood ratio tests. Significant overall tests were followed by pairwise comparisons based on estimated marginal means with Tukey’s adjustments (Lenth 2016). The analyses were performed in R v. 4.1.2 (R Core Team 2021) using the libraries DHARMa (Hartig 2022), emmeans (Lenth 2022), ggplot2 (Wickham 2016), lme4 (Bates *et al.* 2015) and lmerTest (Kuznetsova *et al.* 2017).

## Results

### Chromosome numbers

We confirmed the diploid level as 2*n* = 2*x* = 18 for *D. arbuscula* and both subspecies of *D. cneorum***[see****Supporting Information—Fig. S2A–C****]**. In the presumed polyploid cytotype of *D. cneorum* subsp. *cneorum,* chromosome counting analyses uncovered a triploid cytotype with 2*n* = 3*x* = 27 **[see****Supporting Information—Fig. S2D and Table S1****]**.

### Genome size and ploidy level

**Figure 3.**
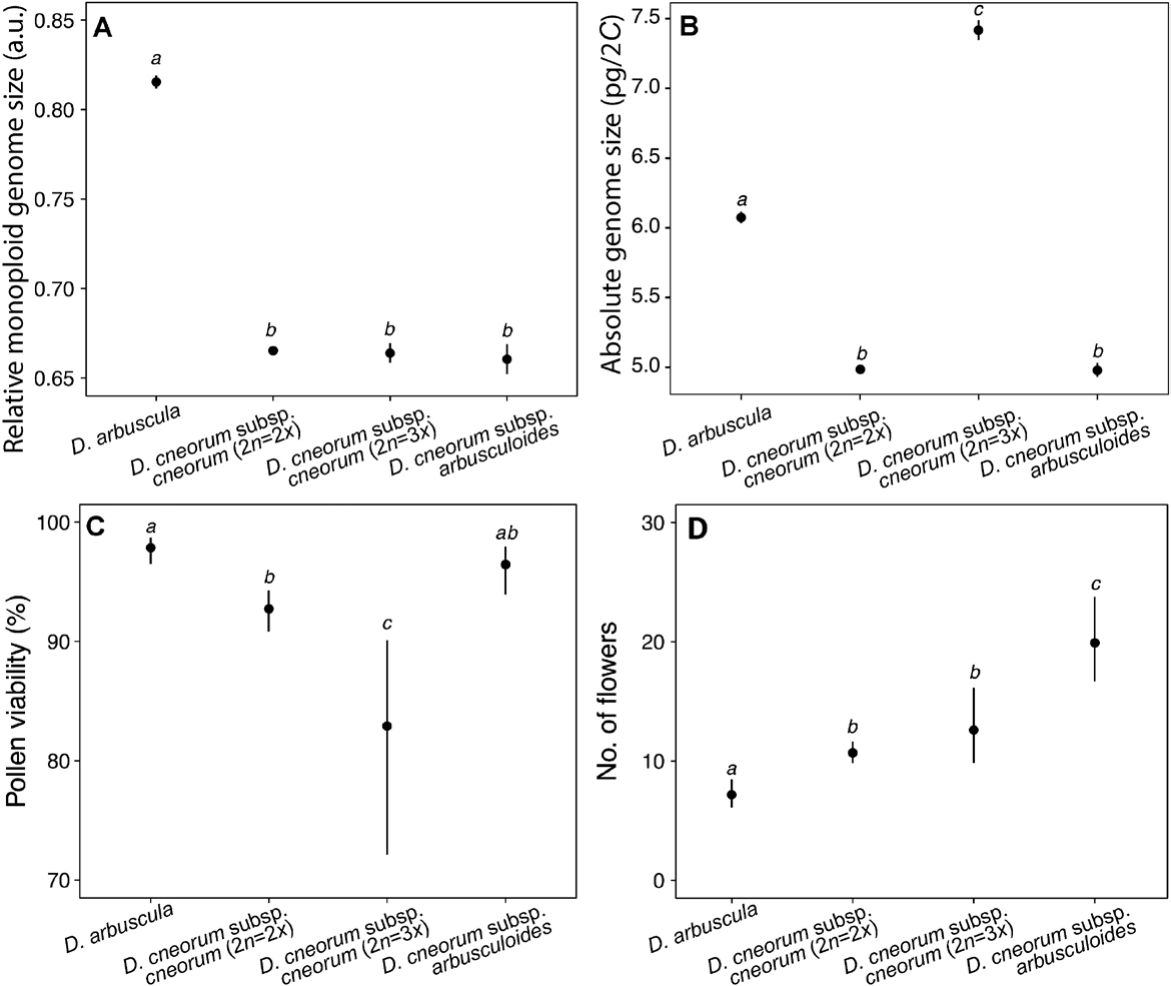
Overall variability in parametric analyses in *Daphne arbuscula*, *D. cneorum* subsp. *cneorum* diploids (2*n* = 2*x*) and triploids (2*n* = 3*x*) and *D. cneorum* subsp. *arbusculoides*: (A) relative monoploid genome size [a.u.]; (B) absolute genome size [pg]; (C) pollen viability [%]; (D) number of flowers per inflorescences. GLMM-estimated mean values (dots) and their 95 % confidence intervals (error bars) are displayed. The estimates labelled with the same lowercase letters were not significantly different according to Tukey’s pairwise comparison, and vice-versa.

Both RGS and AGS analyses showed that all samples were diploid (Table 1) with the exception of five individuals from four different populations of *D. cneorum* subsp. *cneorum* (SK-DC 1, AT-DC 11, HU-DC 17, and RO-DC 19) where both AGS and RGS indicated a triploid ploidy level **[see****Supporting Information—Fig. S3A–B and Table S1****]**. The GLMM revealed significant differences in RGS among investigated taxa (*F*(3, 53.7) = 1485, *P* < 0.0001). *Daphne arbuscula* showed significantly higher RGS than the other taxa whose RGS values were statistically indistinguishable (Fig. 3A; Table 1; **see****Supporting Information—Fig. S3B**). The mean RGS values of *D. cneorum* subsp. *cneorum* DNA-diploids at the monoploid level did not differ statistically from those of DNA-triploids (Fig. 3A; Table 1). Likewise, the monoploid RGS of *D. cneorum* subsp. *arbusculoides* fully overlapped with the RGS of the nominate subspecies (Fig. 3A; Table 1). The intraspecific variation in RGS exceeded 8.59 % in total in the diploid cytotype of *D. cneorum* subsp. *cneorum***[see****Supporting Information—Fig. S4 and Table S1****]**, whereas the interpopulation RGS variability was no more than 5.34 %. However, higher RGS intrapopulation variability was observed, reaching 7.86 % in the SK-DC 1 population **[see****Supporting Information—Table S1****]**. Nevertheless, we were not able to support these RGS differences by using simultaneous analyses. No within- or between-population variation in RGS was found in *D. arbuscula*.

**Table 1.** Relative genome size (RGS), absolute genome size (AGS), GC content, pollen viability, and flower number in studied *Daphne* taxa and cytotypes. Mean, standard deviation (SD), and minimum and maximum values are shown. *Two triploid individuals were analysed.

Taxa/cytotype	RGS euploid (a.u.) ± SD	RGS monoploid (a.u.) ± SD	AGS (pg) ± SD	GC content (%) ± SD	Pollen viability (%)	No. of flowers
*D. arbuscula*	1.63 ± 0.01 (1.61‒1.66)	0.82 ± 0.01 (0.80‒0.83)	6.09 ± 0.03 (6.05‒6.14)	41.42 ± 0.14 (41.22‒41.64)	96.84 ± 3.6 (89‒100)	7.2 ± 1.0 (5‒9)
*D. cneorum* subsp. *cneorum* diploid	1.33 ± 0.02 (1.28‒1.39)	0.66 ± 0.01 (0.64‒0.69)	4.99 ± 0.05 (4.90‒5.11)	41.48 ± 0.32 (40.54‒42.11)	90.78 ± 7.9 (65‒99)	10.9 ± 2.7 (6‒21)
*D. cneorum* subsp. *cneorum* triploid	1.98 ± 0.04 (1.91‒2.04)	0.66 ± 0.01 (0.64‒0.68)	7.38, 7.47*	41.26; 41.47*	79.19 ± 7.2 (66‒91)	12.3 ± 1.7 (9‒14)
*D. cneorum* subsp. *arbusculoides*	1.32 ± 0.01 (1.29‒1.35)	0.66 ± 0.01 (0.65‒0.68)	4.98 ± 0.06 (4.90‒5.12)	41.69 ± 0.24 (41.32‒42.22)	95.93 ± 1.99 (93‒99)	23.0 ± 6.5 (9‒45)

The mean 2*C* values of AGS (Fig. 3B, Table 1) differed significantly among taxa (*F*(3, 17.6) = 2210, *P* < 0.0001). Two analysed triploids of *D. cneorum* subsp. *cneorum* showed significantly higher AGS (7.38 and 7.47 pg) than the other taxa followed by *D. arbuscula* (6.09 ± 0.03 pg), and diploids of *D. cneorum* (4.99 ± 0.05 pg) and *D. cneorum* subsp. *arbusculoides* (4.98 ± 0.06 pg) which had statistically indistinguishable AGS.

The differences in the GC content among the analysed cytotypes and taxa were negligible, with values ranging from 40.54 to 42.22 % (Table 1).

### Pollen viability

Pollen viability was relatively high in all analysed taxa and cytotypes and ranged between 79 and 97 % on average, but it significantly differed among taxa (*χ*^2^(3) = 26.9, *P* < 0.0001). The highest pollen viability was found in *D. arbuscula* (96.84 ± 3.6 %; Fig. 3C; Table 1 and **see****Supporting Information—Table S1**). No significant differences were found between *D. arbuscula* and *D. cneorum* subsp. *arbusculoides* (*z* = 1.35, *P* = 0.53), and between diploid cytotypes of *D. cneorum* subsp. *cneorum* and *D. cneorum* subsp. *arbusculoides* (90.78 ± 7.9 % and 95.93 ± 1.9 %, respectively; *z* = 2.4, *P* = 0.076). The pollen viability of three studied triploid individuals of *D. cneorum* subsp. *cneorum* was high (79.19 ± 7.2 %), but still significantly lower than in the diploid cytotype of *D. cneorum* and *D. arbuscula* (*z* = 2.8, *P* = 0.026).

### Number of flowers in the inflorescences

The number of flowers in inflorescences varied significantly among all three taxa (*χ*^2^(3) = 30.1, *P* < 0.0001). The lowest number of flowers was observed in *D. arbuscula* (7 ± 1.0), while *D. cneorum* subsp. *arbusculoides* had a significantly higher number of flowers (23 ± 6.5) than nominate subspecies *D. cneorum* subsp. *cneorum* (11 ± 2.7). Triploid individuals of *D. cneorum* subsp. *cneorum* did not differ in flower number from diploid *D. cneorum* subsp. *cneorum* (12 ± 1.7; *z* = 1.3, *P* = 0.54; Fig. 3D; Table 1).

### Phylogenetic analyses

**Figure 4.**
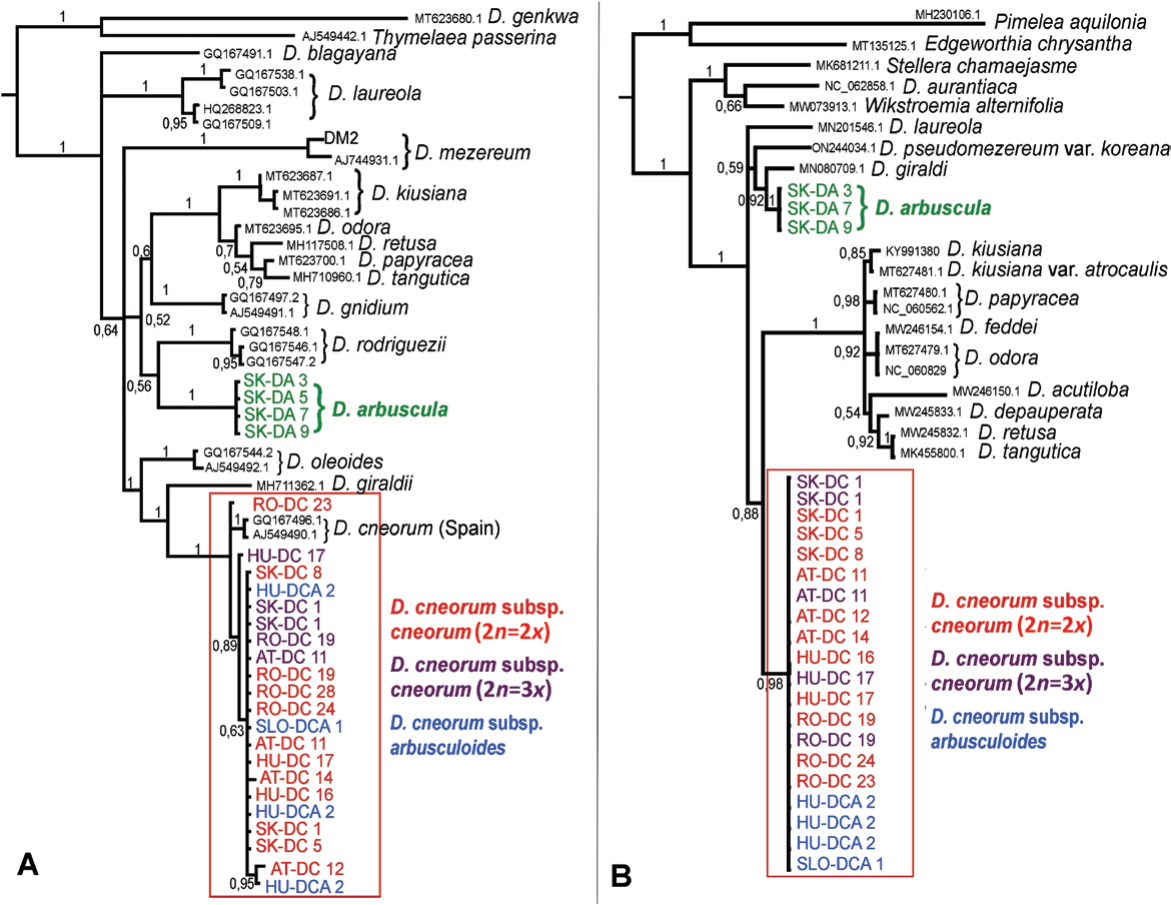
Bayesian inference as recovered by MrBayes: (A) phylogeny based on sequences of the ITS1-5.8S-ITS2 region of nuclear ribosomal DNA; (B) phylogeny based on sequences of the *ndhF-rpl32* region of chloroplast DNA. A combination of sequences generated within this study and those retrieved from GenBank were used in both phylogenies. Bayesian posterior probabilities are indicated near the branches. Population codes of samples generated in this study follow **Supporting Information—Table S1**.

Both BI and ML analyses recovered hierarchically shallow topologies with largely unresolved relationships among the studied taxa in the ITS and plastid phylogenies. We show, however, that *D. arbuscula* and *D. cneorum* are genetically clearly distinct entities, based on both ITS and *ndhF-rpl32* regions (Fig. 4A and B; **see****Supporting Information—Fig. S5A–B**). In the ITS phylogeny, *D. cneorum* appeared clustered with *D. giraldii* and *D. oleoides* (BS = 70, PP = 1). Although *D. arbuscula* forms a strongly supported clade, its relationship with other species remains unresolved (Fig. 4A; **see****Supporting Information—Fig. S5A**). Phylogenetic analyses based on the *ndhF-rpl32* region uncovered an even less resolved topology than ITS; however, *D. arbuscula* and *D. cneorum* formed two well-supported clades (BS = 93, PP = 1, and BS = 99, PP = 0.98, respectively; Fig. 4B; **see****Supporting Information—Fig. S5B**). Both nuclear and plastid data indicate that *D. cneorum* subsp. *arbusculoides* is identical to the nominate subspecies (Fig. 4A and B; **see****Supporting Information—Fig. S5A–B**). The ITS region of the widespread *D. cneorum* subsp. *cneorum* showed minute intraspecific variation; distinct ribotypes were discovered in Spanish and Romanian accessions (RO-DC 23; Fig. 4A; **see****Supporting Information—Fig. S5A**).

## Discussion

### Relict *Daphne* species exhibit extensive karyological stasis

This is the first comprehensive cytogeographic study of this fascinating genus, which has been substantially understudied from a karyological and genetic perspective despite its evolutionary significance due to its relict distribution pattern, and its richness in horticulturally attractive endemic or threatened taxa (Webb and Ferguson 1968; Halda 2001). We uncovered rather limited cytotype variability in all studied taxa; basic diploid ploidy was confirmed in most accessions. However, we found a few triploid individuals from four populations of *D. cneorum* subsp*. cneorum***[see****Supporting Information—Fig. S2D and Table S1****]**. RGS analyses indicate a moderate level of within-species RGS variation in *D. cneorum* subsp*. cneorum* (≤8.59 %), possibly caused by the occurrence of rare aneuploid cytotypes originating from the fusion of reduced and aneuploid gametes. Aneuploid formation appears feasible, especially given the existence of triploids and the fact that diploid individuals with the greatest RGS difference were found in populations with mixed cytotypes (particularly SK-DC 1, **see****Supporting Information—Fig. S4**). However, since we were unable to confirm these minor changes in RGS using simultaneous analyses, we cannot rule out the possibility that at least some of these deviating values were caused by instrumental errors (Greilhuber 1998, 2005) or/and secondary metabolites that caused problems during flow cytometry analyses (cf. Walker *et al.* 2006).

Our results suggest that although *D. cneorum* occurs in small, often highly isolated relict populations along large altitudinal gradients and on various substrates with diverse environmental conditions, its genomes appear to be largely stable, with no extensive genomic rearrangements, or accelerated dynamics in transposable element evolution (Lysák *et al.* 2000; Auckland *et al.* 2001; Bogunic *et al.* 2007; Vít *et al.* 2016). We can only hypothesize about the causes of this genomic stasis in spite of the potential effect of environmental heterogeneity and patchy distribution pattern across its range. One cause could be related to the life strategies of the studied taxa. Even though polyploidization has been found in many woody species, the rate of whole-genome duplication in herbaceous species has been estimated to be about six times higher than in woody species (Zenil-Ferguson *et al.* 2017). Conversely, long-lived species may have more opportunities for somatic duplication than short-lived taxa (Stebbins 1938; Grant 1981), and the formation of unreduced gametes and their successful fusion with (un)reduced gametes may be more common in species reproducing regularly over multiple years (Otto and Whitton 2000). Similarly, in the case of *D. cneorum*, the survival and persistence of triploid cytotypes in natural populations are likely to be facilitated by a longer lifespan, typical of woody perennials with clonal growth (Lynch *et al.* 1998; Nakagawa *et al.* 1998; Joly and Bruneau 2004; Truong *et al.* 2015; Morgan *et al.* 2021). In fact, clonality can contribute to the survival and vegetative reproduction of these triploids, albeit to a limited extent. In contrast, rare cytotypes, including those with an odd ploidy level in annual or short-lived species, have ephemeral persistence and rely on recurrent *de novo* formation (e.g. Čertner *et al.* 2017).

### Occurrence and origin of triploid cytotypes in *D. cneorum*

While polyploidy has not played a prominent role in the evolutionary history of the genus *Daphne* (Bolkhovskikh *et al.* 1969; Murín 1990; Dawson and Beuzenberg 2013), several instances of autopolyploids and allopolyploids have been reported (Malla *et al.* 1977; Landolt and Hauser 1981; Urbani 1992). Our study reveals a unique example of a triploid cytotype reoccurring in several populations of *D. cneorum* in central Europe. Exceptional cases of triploid cytotypes have already been detected in *D. mezereum* (Pustahija *et al.* 2013) and *D. odora* Thunb. (2*n* = 27, 28, 30; Osawa 1913; Yamaha 1927; Takenaka 1931; Hiraoka 1958; Okura and Kono 1959). Triploid cytotypes may form either as a result of the fusion of reduced and unreduced gametes in purely diploid populations or heteroploid hybridization between predominantly diploid and tetraploid cytotypes (Ramsey and Schemske 1998; Husband *et al.* 2013). The following observations in our study lend support to the former hypothesis: (i) No tetraploid cytotypes were found in any of the studied populations to suggest heteroploid gene flow and the formation of triploid cytotypes. Although we cannot definitively rule out the existence of an unsampled tetraploid cytotype in *D. cneorum,* it is not very likely as polyploid cytotypes have never been detected in this species (Goldblatt and Johnson 1979; Rice *et al.* 2015; **see****Supporting Information—Table S2**). In addition, if triploids would result from heteroploid hybridization, indicating the presence of a triploid bridge between cytotypes (Kolář *et al.* 2017), we would anticipate a higher prevalence of triploid cytotypes in given populations (cf. Burton and Husband 2001; Čertner *et al.* 2017); (ii) The monoploid genome size of diploids and triploids were identical (Fig. 3A), indicating that they most likely originated from the same parental taxon; (iii) The triploids were morphologically similar to their diploid counterparts and even had the same number of flowers per inflorescence (Figs. 1D, E and 3D), ruling out the possibility of hybridization with another related, but morphologically distinct polyploid taxon; (iv) Likewise, the results of our phylogenetic analyses do not indicate the presence of other genomes than that of *D. cneorum* in detected triploid cytotypes (Fig. 4A and B; **see****Supporting Information—Fig. S5A–B**). Although none of these observations explicitly reject the allopolyploid origin of triploids in *D. cneorum*, the combination of factors mentioned above makes this alternative hypothesis highly improbable. Furthermore, the detected triploids likely evolved independently because they appear in different, often geographically distant, populations with diverse environmental conditions, that is, various altitudes and bedrock types (Figs. 1F–I and 2; **see****Supporting Information—Table S1**).

### Pollen viability of triploids in *D. cneorum* and their frequency and persistence in diploid populations

Triploids in sexually reproducing species have typically been regarded as a dead end due to low fertility caused by irregular chromosomal pairing and unequal meiotic division, resulting in the formation of a range of euploid and aneuploid gametes (Ramsey and Schemske 1998; Husband 2004). Unbalanced chromosomal compositions of triploids and aneuploids typically lead to sterility (Eckert 2001; Husband 2004). Irregularities in meiosis and aneuploid gamete production were demonstrated, for instance, in the Asian congener, *D. odora* (Osawa 1913; Yamaha 1927; Takenaka 1931; Hiraoka 1958; Okura and Kono 1959). In contrast to *D. odora* (Osawa 1913), however, the pollen viability in triploids and diploids of *D. cneorum* is comparable, ranging from 66 to 91 % (Fig. 3C; Table 1). This indicates that triploids in *D. cneorum* subsp. *cneorum* could theoretically be considered at least semi-fertile due to their high pollen viability (Schinkel *et al.* 2017). The pollen fertility of triploids in angiosperms is highly variable and species-specific, ranging from 0 to 97 % (Ramsey and Schemske 1998). Low pollen viability was detected, for instance, in triploid hybrid individuals of *Populus* (2.78 %; Wang *et al.* 2017), *Betula* (8–9 %; Anamthawat-Jónsson *et al.* 2021), and *Tamarix* cf*. kermanensis* (28.5 %; Samadi *et al.* 2011). In contrast, triploids of *Arabidopsis arenosa* can produce even 82 % viable pollen grains (Morgan *et al.* 2021). The pollen viability revealed in both cytotypes and subspecies of *D. cneorum* and *D. arbuscula* is markedly higher than that of their congeners, *D. gnidium* (41 %; Roccotiello *et al.* 2009), *D. genkwa* (51 %; Liu *et al.* 2011) and *D. aurantiaca* (35–75 %; Liu *et al.* 2018). Our results are also not in line with the assumption that low pollen viability might be a potential factor responsible for the low success of fruit production in *D. arbuscula* and *D. cneorum* (Erdelská and Turis 1995; Gajdošová 2020).

The frequency of *D. cneorum* triploids in three populations varied between 0.71 and 1.3 %, whereas in population RO-DC 19 this incidence was 10 %. Although bedrock type was not indicative of the occurrence of triploid individuals, most populations harbouring odd cytotypes are characterized by harsh drought conditions (RO-DC 19 on gypsum, SK-DC 1 on siliceous sands, or HU-DC 17 on the calcareous substrate; Fig. 1G–I; **see****Supporting Information—Table S1**). We may hypothesize that extreme microclimate conditions could act as a major environmental driver in shifting survival strategies and promoting clonality (e.g. Ramsey and Schemske 1998; Mock *et al.* 2012). In addition, it is known that the formation of unreduced gametes is more frequent under stressful conditions, which in turn can lead to an increase in triploid cytotype formation in woody perennials (e.g. Harlan and deWet 1975; Ramsey and Schemske 1998). More data on both *in situ* microclimate particularities and the extent of clonality in these populations are required in order to fully test this hypothesis. Conversely, this result is also attributable to the small sample sizes belonging to these populations **[see****Supporting Information—Table S1****]**. Comparatively low frequencies of triploid individuals within diploid populations have also been reported in other woody species, specifically 2.8 % of triploids in *Olea europaea* (Besnard and Baali-Cherif 2009) and from 0.25 to 0.57 % in various *Quercus* species (Dzialuk *et al.* 2007 and citations therein). There are multiple explanations for the rarity of triploids in sexually reproducing diploid plant species. In fact, their absence may be due to the very low incidence of unreduced gametes, with production in native populations rarely exceeding 2 %. It is reported to be higher in asexual species than in sexual species with selfing, mixed mating, or outcrossing pollination (Ramsey 2007; Kreiner *et al.* 2017). Even after the successful fusion of 2*n* and *n* gametes, seeds could have significantly reduced viability and germination abilities (Burton and Husband 2000; Truong *et al.* 2015; Wang *et al.* 2016). Furthermore, even after successful germination (e.g. Rounsaville *et al.* 2011), their fitness and competitiveness in the environment in a given population can be weak (Burton and Husband 2000; Baack 2005; Truong *et al.* 2015; Morgan *et al.* 2021), and they may not survive to the stage of a fully developed, reproducible individual (Truong *et al.* 2015; Wang *et al.* 2016). Our study calls for further research to determine whether triploids in *D. cneorum* produce their own seeds, their viability, and whether potential seedlings survive to the reproductive stage.

### Notes on evolutionary relationships and taxonomy of studied *Daphne* taxa

Phylogenetic analyses revealed that *D. cneorum* and *D. arbuscula* are well-defined species. Even though the overall relationships between the taxa were only partially resolved, there appears to be little support for the previously predicted close relationship between *D. arbuscula* and *D. cneorum* (Tuzson 1911; Halda 1976, 2001). Intriguingly, *D. cneorum* subsp. *arbusculoides*, which has been reported as separate endemic subspecies within *D. cneorum*, cannot be distinguished from nominate subspecies based on ITS and cpDNA sequences or karyological traits like ploidy and genome size. As reported in taxonomic literature, both subspecies should differ not only in terms of ecology and distribution range but also with respect to morphological traits (Tuzson 1911; Webb and Ferguson 1968; Soó 1971; Halda 1976). Our study shows that *D. cneorum* subsp. *arbusculoides* accessions differ significantly in flower number from those of *D. cneorum* subsp. *cneorum*. Another important diagnostic trait delimiting this taxon should be a more ascending habitus and the shape of the leaf margins, which we did not formally test here. To draw sound taxonomic conclusions, further taxonomic research using a combination of morphological, ecological, and robust molecular markers (NGS-based) analyses is necessary.

## Supporting Information

The following additional information is available in the online version of this article –

**Table S1.** Details of localities, genome size values, chromosome numbers, pollen viability, and number of flowers in inflorescences for sampled populations of *Daphne* taxa from the present study.

**Table S2**. A synthesis of published chromosome numbers and genome size values within the genus *Daphne.*

**Figure S1**. Stained pollen grains of studied *Daphne* taxa considered as viable (magenta–red) and non-viable (blue–green).

**Figure S2**. Microphotographs of chromosome mitotic metaphase of studied *Daphne* taxa.

**Figure S3**. Differences in RGS of studied *Daphne* taxa.

**Figure S4**. Boxplots of RGSs for populations of studied *Daphne* taxa.

**Figure S5**. Maximum likelihood analyses as recovered by RaxML.

plad056_suppl_Supplementary_Material

## Data Availability

Data are provided as Supporting Information. The datasets generated for this study can be found in online repositories (https://www.ncbi.nlm.nih.gov/genbank/) with GenBank accession numbers OQ269406–OQ269431 for 26 ITS sequences and OQ320711–OQ320733 for 23 cpDNA sequences (ndhF–rpl32 region).

## Notes

**Evolution & Diversity. Section Editor:** Jeremy Beaulieu
