# Supplementary material for: Incidence and evolutionary relevance of autotriploid cytotypes in a relict member of the genus Daphne (Thymelaeaceae)
Source: AoB Plants. 2023 Aug 30;15(5):plad056. doi: 10.1093/aobpla/plad056 (PMC10601019; doi:10.1093/aobpla/plad056)
Supplement: plad056_suppl_Supplementary_Material [file plad056_suppl_supplementary_material.pdf]

Supporting Information

AoB Plants

Incidence and evolutionary relevance of autotriploid cytotypes in a relict member of the genus *Daphne* (Thymelaeaceae)

Zuzana Gajdošová[1], Marek Svitok[2,3], Veronika Cetlová[1], Lenka Mártonfiová[4], Jaromír Kučera[1], Vladislav Kolarčik[5], Bogdan-Iuliu Hurdu[6], Ioana-Minodora Sîrbu[7], Ingrid Turisová[8], Peter Turis[8] and Marek Slovák[1,9]

[1] *Plant Sciences and Biodiversity Centre, Slovak Academy of Sciences, Dúbravská cesta 9, SK-845 23 Bratislava, Slovak Republic*

[2] *Department of Biology and General Ecology, Faculty of Ecology and Environmental Sciences, Technical University in Zvolen, Ul. T. G. Masaryka 24, SK-960 01 Zvolen, Slovak Republic*

[3] *Department of Forest Ecology, Czech University of Life Sciences Prague, CZ-16 521 Suchdol, Praha 6, Czech Republic*

[4] *Botanical Garden of Pavol Jozef Šafárik University in Košice, Mánesova 23, SK-043 52, Košice, Slovak Republic*

[5] *Institute of Biology and Ecology, Faculty of Science, Pavol Jozef Šafárik University in Košice, Mánesova 23, SK-041 54, Košice, Slovak Republic*

[6] *Institute of Biological Research, 48 Republicii St., R-400015 Cluj-Napoca, Romania*

[7] *Faculty of Biology, University of Bucharest, Splaiul Independenței 91–95, R-050095 Bucharest, Romania*

[8] *Department of Biology and Ecology, Faculty of Natural Sciences, Matej Bel University in Banská Bystrica, Tajovského 40, SK-974 01 Banská Bystrica, Slovak Republic*

[9] *Department of Botany, Faculty of Science, Charles University, Benátská 2, CZ-128 01 Praha, Czech Republic*

Supporting Information_Figures

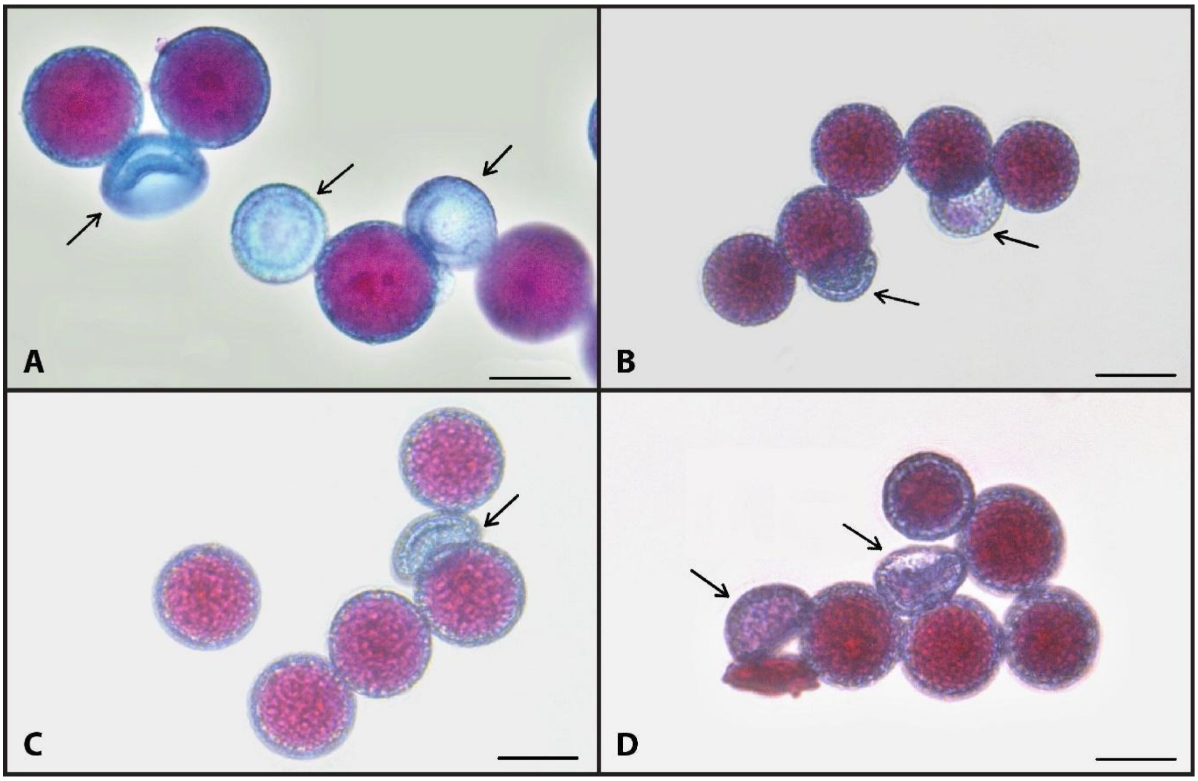

Supporting Information Figure S1. Stained pollen grains of studied *Daphne* taxa considered as viable (magenta-red) and non-viable (blue-green): (A) *Daphne arbuscula*; (B) *D. cneorum* subsp. *arbusculoides*; (C) *D. cneorum* subsp. *cneorum* diploid; (D) *D. cneorum* subsp. *cneorum* triploid. The arrow indicates non-viable pollen grains; scale bar = 20 µm.

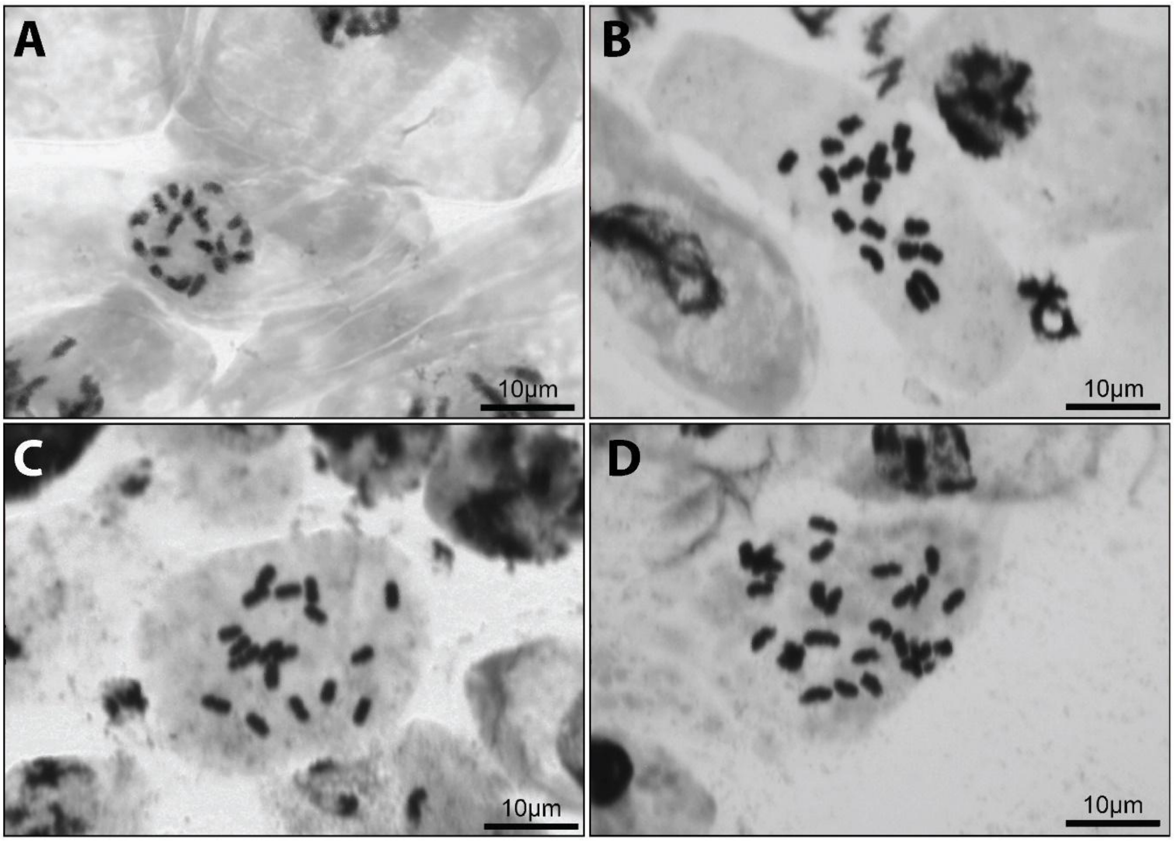

Supporting Information Figure S2. Microphotographs of chromosome mitotic metaphase of studied *Daphne* taxa: (A) *Daphne arbuscula* (SK-DA 9), 2*n* = *2x* = 18; (B) *D. cneorum* subsp. *arbusculoides* (HU-DCA 2), 2*n* = *2x* = 18; (C) *D. cneorum* subsp. *cneorum* (SK-DC 8), 2*n* = *2x* = 18; (D) *D. cneorum* subsp. *cneorum* (SK-DC 1) 2*n* = *3x* = 27; scale bar = 10 µm.

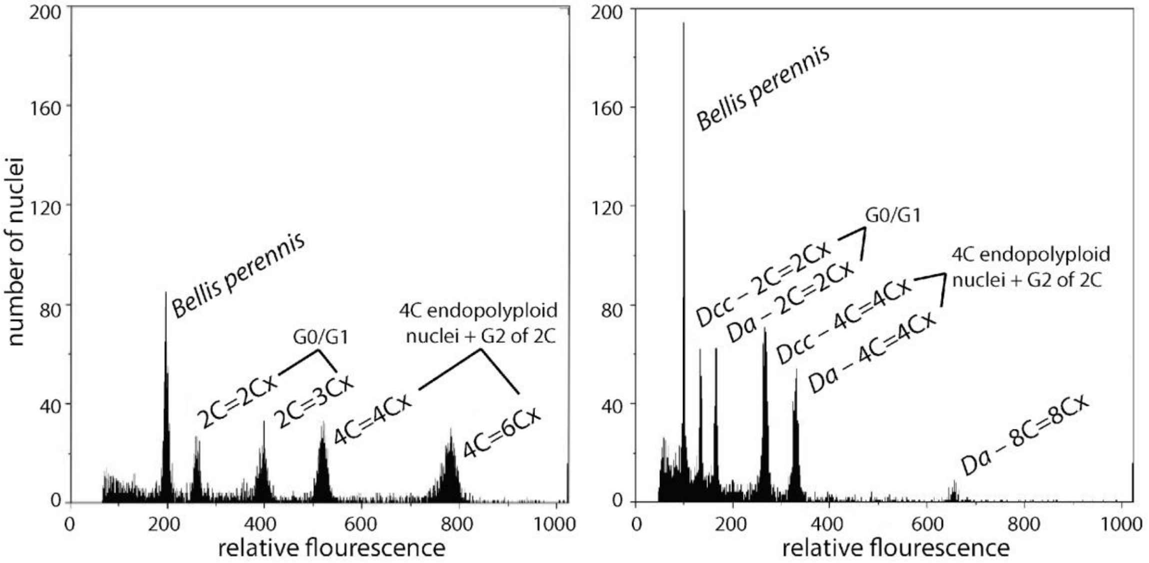

Supporting Information Figure S3. Differences in relative genome size of studied *Daphne* taxa: (A) diploid and triploid individuals of *D. cneorum* subsp. *cneorum*; (B) diploid individuals of *Daphne arbuscula* and *D. cneorum* subsp. *cneorum*. Nuclei from all individuals were isolated, stained with DAPI and analysed simultaneously.

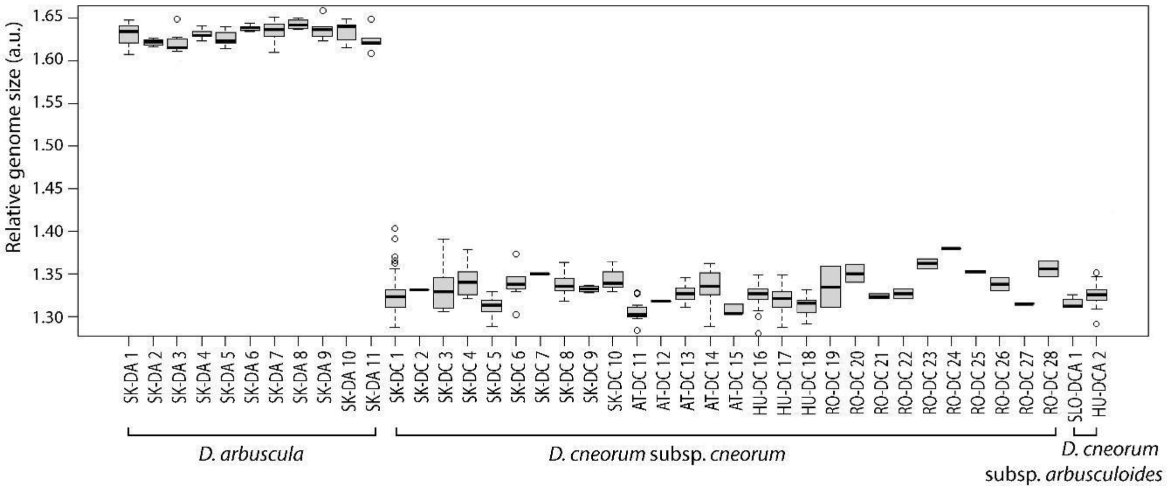

Supporting Information Figure S4. Boxplots of relative genome sizes for populations of studied *Daphne* taxa. Minimum, first quartile, median (thick line), third quartile, maximum, and outlier values (dots) are shown. Population codes of samples follow Supporting Information—Table S1.

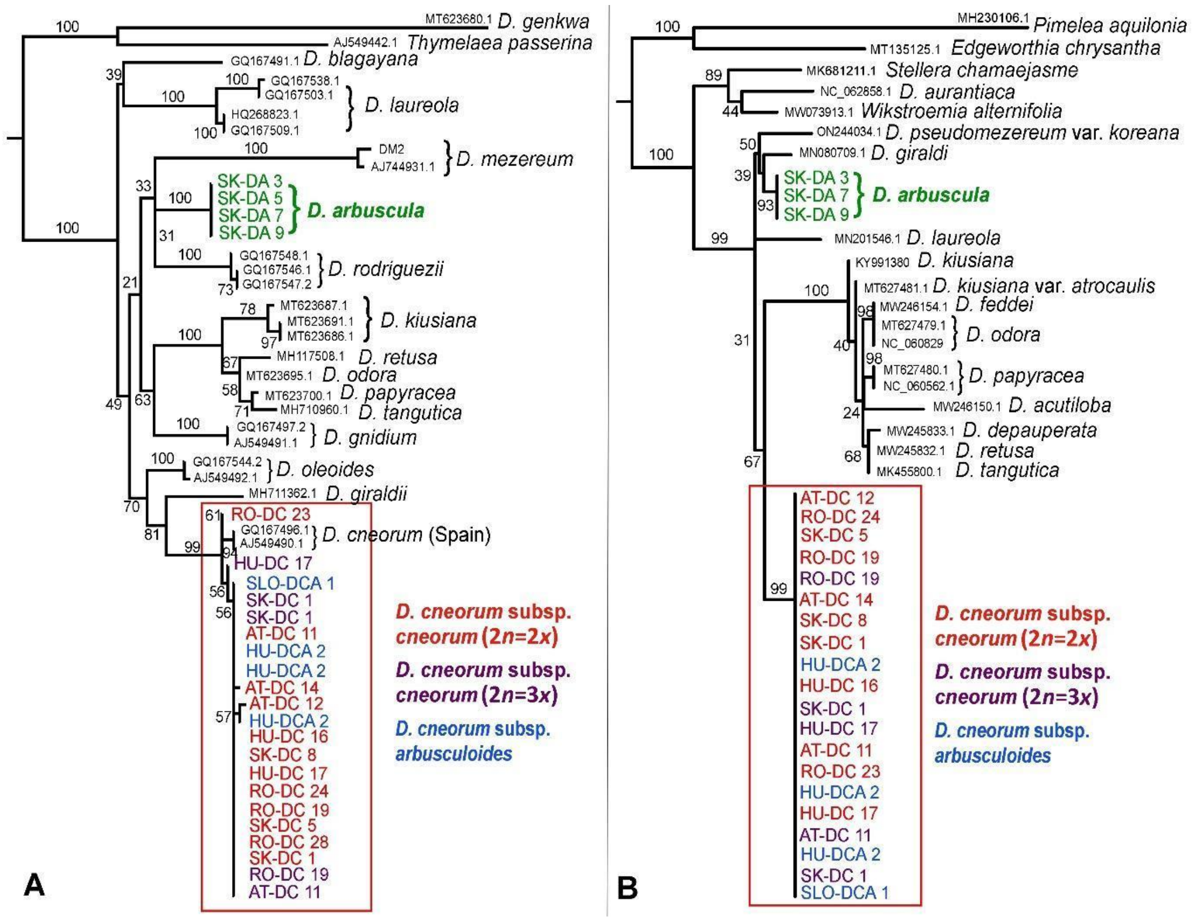

Supporting Information Figure S5. Maximum likelihood analyses as recovered by RaxML: (A) phylogeny based on sequences of the ITS1-5.8S-ITS2 region of nuclear ribosomal DNA; (B) phylogeny based on sequences of the *ndhF-rpl32* region of chloroplast DNA. A combination of sequences generated within this study and those retrieved from GenBank were used in both phylogenies. Bootstrap values are indicated near the branches. Population codes of samples generated in this study follow Supporting Information—Table S1.

Supporting Information Table S1. Details of localities, genome size values, chromosome numbers, pollen viability, and number of flowers in inflorescences for sampled populations of *Daphne* taxa from the present study. Abbreviations: RGS – relative genome size based on DAPI measurements [a.u.]; AGS – absolute genome size 2*C* DNA values [pg]; CN – chromosome number; N – number of analysed individuals per population; SD – standard deviation. Collector abbreviations: AI – Adrian Indreica, BIH – Bogdan-Iuliu Hurdu, DB – Drahoš Blanár, EGŠ – Eliška Gbúrová Štubňová, IMS – Ioana-Minodora Sîrbu, IT – Ingrid Turisová, JK – Jaromír Kučera, MS – Marek Slovák, PT – Peter Turis, ZG – Zuzana Gajdošová.

ID	Locality	Bedrock	RGS ± SD (N)	AGS ± SD [pg] (N)	CN (2*n*)	Pollen viability ± SD [%] (N)	No. of flowers ± SD (N)
Daphne arbuscula							
SK-DA 1	Slovakia, Western Carpathians, Muránska Planina Mts., Kášter, 910 m a.s.l., coll.: DB, JK, MS, PT, ZG	calcareous	1.63 ± 0.02 (7)	6.09 ± 0.04 (3)	-	-	-
SK-DA 2	Slovakia, Western Carpathians, Muránska Planina Mts., Hradová, 870 m a.s.l., coll.: DB, ZG	calcareous	1.62 ± 0.00 (7)	-	-	-	-
SK-DA 3	Slovakia, Western Carpathians, Muránska Planina Mts., Malá Stožka, 1170 m a.s.l., coll.: MS, ZG	calcareous	1.62 ± 0.01 (10)	-	-	91.25 ± 2.36 (2)	6.8 ± 1.1 (10)
SK-DA 4	Slovakia, Western Carpathians, Muránska Planina Mts., Šarkanica, 970 m a.s.l., coll.: MS, ZG	calcareous	1.63 ± 0.01 (5)	-	-	-	-
SK-DA 5	Slovakia, Western Carpathians, Muránska Planina Mts., Veľká Stožka, 1275 m a.s.l., coll.: MS, ZG	calcareous	1.63 ± 0.01 (10)	-	-	96.60 ± 3.56 (6)	7.5 ± 0.7 (10)

SK-DA 6	Slovakia, Western Carpathians, Muránska Planina Mts., Hrdzavá dolina – Vrbjarka, 1085 m a.s.l., coll.: DB, IT, ZG	calcareous	1.64 ± 0.00 (5)	-	-	-	-
SK-DA 7	Slovakia, Western Carpathians, Muránska Planina Mts., Poludnica – Klin, 790–830 m a.s.l., coll.: DB, JK, MS, PT, ZG	calcareous	1.64 ± 0.01 (20)	6.10 ± 0.04 (3)	-	98.80 ± 2.40 (5)	7.5 ± 0.8 (10)
SK-DA 8	Slovakia, Western Carpathians, Muránska Planina Mts., Cigánka, 880 m a.s.l., coll.: JK, MS, ZG	calcareous	1.64 ± 0.01 (5)	-	-	-	-
SK-DA 9	Slovakia, Western Carpathians, Muránska Planina Mts., Šiance, 950 m a.s.l., coll.: DB, JK, MS, PT, ZG	calcareous	1.64 ± 0.01 (10)	6.08 ± 0.01 (3)	18	97.40 ± 2.65 (5)	7.0 ± 1.2 (10)
SK-DA 10	Slovakia, Western Carpathians, Muránska Planina Mts., Havrania dolina – Slaniniarka, 1033 m a.s.l., coll.: IT, ZG	calcareous	1.63 ± 0.01 (5)	-	-	-	-
SK-DA 11	Slovakia, Western Carpathians, Muránska Planina Mts., Zlatnica – Čupková, 950 m a.s.l., coll.: IT, ZG	calcareous	1.62 ± 0.01 (5)	-	-	-	-
Daphne cneorum subsp. *cneorum*							
SK-DC 1	Slovakia, Pannonian basin, Záhorská Nížina lowland, Červený kríž 180 m a.s.l., coll.: JK, MS, ZG	siliceous sands	1.32 ± 0.02 (222)<br>1.98 ± 0.04 (2)	5.00 ± 0.02 (3)<br>7.43 ± 0.11 (2)	-<br>27	94.55 ± 1.44 (7)<br>85.18 ± 3.70 (1*)	10.25 ± 2.0 (20)<br>9 (1)
SK-DC 2	Slovakia, Western Carpathians, Považský Inovec Mts., Kňaží stôl, 586 m a.s.l., coll.: JK, EGŠ	calcareous	1.33 (1)	-	-	-	-
SK-DC 3	Slovakia, Western Carpathians, Strážovské Vrchy Mts., Stupičie, 685 m a.s.l., coll.: JK, EGŠ	calcareous	1.33 ± 0.03 (20)	4.99 ± 0.03 (4)	-	-	8.8 ± 1.7 (10)

SK-DC 4	Slovakia, Western Carpathians, Žiar Mts., near Ráztočno village, 590 m a.s.l., coll.: JK, MS	calcareous	1.34 ± 0.02 (15)	5.08 ± 0.02 (3)	-	-	-
SK-DC 5	Slovakia, Western Carpathians, Veľká Fatra Mts., Ostrá, 1240 m a.s.l., coll.: IT, PT, ZG	calcareous	1.31 ± 0.01 (16)	4.99 ± 0.00 (3)	-	85.23 ± 13.55 (5)	-
SK-DC 6	Slovakia, Western Carpathians, Veľká Fatra Mts., Sokol, 610 m a.s.l., coll.: PT, ZG	calcareous	1.34 ± 0.02 (22)	4.97 ± 0.03 (3)	-	-	-
SK-DC 7	Slovakia, Western Carpathians, Chočské vrchy Mts., Čebrať, 775 m a.s.l., coll.: PT, ZG	calcareous	1.35 (3)	4.97 ± 0.01 (3)	-	-	-
SK-DC 8	Slovakia, Western Carpathians, Kozie Chrbty Mts., Baba, 780 m a.s.l., coll.: ZG	calcareous	1.35 ± 0.01 (25)	4.93 ± 0.02 (3)	18	84.66 ± 7.15 (5)	11.1 ± 1.3 (10)
SK-DC 9	Slovakia, Western Carpathians, Slovenský Raj Mts., Ihrík, 650 m a.s.l., coll.: ZG	calcareous	1.33 ± 0.00 (12)	4.96 ± 0.07 (3)	-	-	-
SK-DC 10	Slovakia, Western Carpathians, Branisko Mts., Rajtopíky, 870 m a.s.l., coll.: ZG	calcareous	1.34 ± 0.02 (9)	4.97 ± 0.02 (3)	-	-	-
AT- DC 11	Austria, Eastern Alps Mts., Hochschwab Mts., Karlschutt, 870 m a.s.l., coll.: JK, ZG	calcareous	1.31 ± 0.01 (76)<br>1.91 (1)	-<br>-	-<br>-	95.07 ± 3.64 (5)<br>-	11.9 ± 2.8 (20)<br>-
AT- DC 12	Austria, Pannonian Basin, Güns Mts., Jobst, 290 m a.s.l., coll.: JK, MS, ZG	siliceous	1.32 (4)	-	-	-	-
AT- DC 13	Austria, Eastern Alps Mts., Wienerwald Mts., Perchtoldsdorfer Heide, 325 m a.s.l., coll.: JK, MS, ZG	calcareous	1.33 ± 0.01 (33)	-	-	-	-
AT- DC 14	Austria, Pannonian Basin, Little Hungarian Plain, Siegendorfer Puszta, 195 m a.s.l., coll.: JK, ZG	loess	1.34 ± 0.02 (247)	-	-	94.00 ± 3.19 (5)	11.75 ± 2.8 (20)
AT- DC 15	Austria, Pannonian Basin, Marchfeld, Weikendorfer Remise, 150 m a.s.l., coll.: JK, ZG	siliceous sands	1.31 ± 0.01 (23)	-	-	-	-

HU- DC 16	Hungary, Pannonian Basin, Little Hungarian Plain, Gönyű, 120 m a.s.l., coll.: JK, ZG	calcareous	1.32 ± 0.01 (155)	-	-	93.59 ± 4.90 (5)	13.7 ± 3.5 (20)
HU- DC 17	Hungary, Pannonian Basin, Transdanubian Mts.,Vértes Mts., Gánt, 240 m a.s.l., coll.: JK, ZG	calcareous	1.33 ± 0.13 (140)<br>1.96 (1)	-<br>-	-<br>-	92.29 ± 3.11 (5)<br>70.50 ± 3.27 (1*)	9.8 ± 1.7 (20)<br>12.7 ± 0.9 (1*)
HU- DC 18	Hungary, Pannonian Basin, Transdanubian Mts., Tetényi-fennsik, 220 m a.s.l., coll.: JK, ZG	calcareous	1.31 ± 0.01 (29)	-	-	-	-
RO- DC 19	Romania, Transylvania, Transylvanian Plateau, Almaș Depression, Sfãraş-Jebucu, 419 m a.s.l., coll.: BIH, IMS	gypsum	1.33 ± 0.02 (10)<br>2.02 (1)	-<br>-	-<br>-	88.65 ± 9.42 (5)<br>81.89 ± 3.78 (1*)	11.22 ± 1.0 (9)<br>13 (1)
RO- DC 20	Romania, Apuseni Mts., Gilău-Muntele Mare Mts., Scărița-Belioara, 1365 m a.s.l., coll.: BIH, IMS	calcareous	1.35 ± 0.01 (10)	-	-	-	-
RO- DC 21	Romania, Apuseni Mts., Trascău Mts., Turda Gorge, ”La Dos”, 674 m a.s.l., coll.: BIH, IMS	calcareous	1.32 ± 0.00 (10)	-	-	-	10.2 ± 1.8 (10)
RO- DC 22	Romania, Transylvania, Someș Plateau, Valea Gârbăului, 458 m a.s.l., coll.: BIH, IMS	calcareous	1.33 ± 0.01 (10)	-	-	-	-
RO- DC 23	Romania, Southern Carpathians, Retezat Mts., Piule Mt., 1796 m a.s.l., coll.: BIH, IMS	calcareous	1.36 ± 0.01 (10)	-	-	-	8.67 ± 1.9 (9)
RO- DC 24	Romania, Southern Carpathians, Cozia Mts., Doabra peak, Pădurea Călinești Nature Reserve, 818 m a.s.l., coll.: BIH, IMS	conglomerate and sandy stone	1.38 (6)	-	-	89.03 ± 9.71 (5)	10.0 ± 2.1 (5)
RO- DC 25	Romania, Southern Carpathians, Piatra Craiului Mt., Padina Hotarului, below Turnu peak, 1790 m a.s.l., coll.: AI	calcareous	1.35 ± 0.00 (10)	-	-	-	-

RO- DC 26	Romania, Eastern Carpathians, Giurgeu Mts., Bizac gorges, below Suhardul Mic peak, 1205-1342 m a.s.l., coll.: BIH, IMS	calcareous	1.34 ± 0.01 (11)	-	-	-	-
RO- DC 27	Romania, Eastern Carpathians, Ciuc Mts., above Delnița village, 850 m a.s.l., coll.: BIH, IMS	calcareous	1.31 ± 0.00 (10)	-	-	-	-
RO- DC 28	Romania, Eastern Carpathians, Ciuc Mts., above Belani village, 800-928 m a.s.l., coll.: BIH, IMS	flysch	1.36 ± 0.01 (10)	-	-	94.81 ± 3.07 (5)	10.4 ± 1.5 (5)
Daphne cneorum subsp. *arbusculoides*							
SLO-DCA 1	Slovenia, Pannonian Basin, Western Hungarian Borderland, Goričko region, Hodoš, 260 m a.s.l., coll.: JK, MS, ZG	siliceous	1.31 ± 0.01 (19)	5.01 ± 0.06 (5)	-	95.85 ± 2.43 (5)	15.47 ± 4.1 (15)
HU-DCA 2	Hungary, Pannonian Basin, Western Hungarian Borderland, Őrseg region, Szalafő, 240 m a.s.l., coll.: JK, MS, ZG	siliceous	1.33 ± 0.01 (158)	4.95 ± 0.04 (5)	18	96.01 ± 1.42 (5)	24.44 ± 5.9 (80)

* multiple samples from the same individual were studied to increase the accuracy of the analysis

Supporting Information Table S2. A synthesis of published chromosome numbers and genome size values within the genus *Daphne*. Abbreviations: CN – chromosome number [(2*n*) euploid and (*n*) haploid]; 2*C* – absolute genome size value of a euploid genome; 1*C* –absolute genome size value of a monoploid genome. Asterisks indicate the chromosome number from an unverified source. Novel data obtained in the present study are highlighted in bold.

Published as	CN (2*n*)	CN (*n*)	2*C* (pg)	1*C* (pg)	Country and source
D. alpina L. (syn. *D. alpina* L. subsp. *alpina*)			8.17	4.09	Croatia, Mt. Biokovo: Siljak-Yakovlev *et al*. 2010
	18	9			Indonesia, Java, Buitenzorg Botanic Gardens (cult.): Strasburger 1909
	36				Italy, Rupi di Loreto Triora, Liguria: Urbani 1992
***D. arbuscula* Čelak.**	18				Slovakia, Muránska Planina Mts., Mochnatá: Murín in Májovský *et al*. 1978
	18				Slovakia, Muránska Planina Mts., Mochnatá: Murín 1990
	**18**		**6.08**	**3.04**	**Slovakia, Muránska Planina Mts., Šiance: present study**
D. blagayana Freyer			4.70	2.35	Bosnia and Herzegovina, Kladanj: Siljak-Yakovlev *et al*. 2010; Bosnia and Herzegovina, Kladanj: Pustahija *et al*. 2013
	18				Greece, Mt. Olympus: Van Loon in Löve 1982
	18				Yugoslavia: Nevling *et al.* 1962
	18	9			unknown locality (cult.): Blaise 1959
D. burkwoodii Turrill		9			USA, Harvard, Arnold Arboretum (cult.): Nevling in Löve and Solbrig 1964
***D. cneorum* L. (syn. *D. cneorum* L. subsp. *cneorum*)**		9			Austria, Hoher Lindkogel bei Baden: Fuchs 1938
	18				Bulgaria, Pirin Mts., Bajuva Dupka: Nikolov 1991
	18	9			Czech Republic, Mělník: Krahulcová 1991

	18		4.75	2.37	Czech Republic, Moravia, Hrotovice: Šmarda *et al*. 2019
	18				Hungary, the Solymár wall; Cseresnyésárok, Szár: Baksay 1956
	18				Poland, Reservation Krzemionki near Ostrowiec Świętokrzyski: Wcisło in Pogan *et al*. 1990
	18				Slovakia, Slovenský raj, Ihrík: Uhríková and Májovský in Löve 1978
	**18**		**4.93**	**2.47**	**Slovakia, Kozie chrbty Mts., Baba: present study**
	**27**		**7.38**	**3.69**	**Slovakia, Záhorská nížina Lowland: present study**
***D. cneorum* subsp. *arbusculoides* (Tuzson) Soó**	**18**		**4.95**	**2.48**	**Hungary, Őrseg region, Szalafő: present study**
D. genkwa Siebold & Zucc.		9			USA, Harvard, Arnold Arboretum (cult.): Nevling 1962
D. giraldii Nitsche		9			USA, Harvard, Arnold Arboretum (cult.): Nevling 1962
D. glomerata Lam.	18				Caucasus: Sokolovskaya and Strelkova 1940
D. gnidium L.		9			France, Oléron Island: Delay 1969
		9			Spain, Sevilla, El Garrobo: Pastor *et al*.1988
		9			Spain, Huelva: Diosdado *et al*.1993
	18				Algeria, Setif region: Ramdani *et al*. 2015
	18				France, Montpellier: Natarajan in Löve 1978
	18				Italy, Sauci Piccolo, Sauci Vito Lo Capo: Romano *et al*. 1986
	18				Portugal, Lisabon: Blaise 1959
	18				Spain, Malaga, Fuengirola: Bjorkqvist *et al*. 1969
D. jasminea Sibth. & Sm.		9			Greece, Crete, Selinos, Gorges d'Aghia Irini: De Montmollin 1986
D. jezoensis Maxim. (syn. *D. kamtschatica* var. *jezoensis* (Maxim.) Ohwi)	18				Japan, Mt. Teine, prov. Ishikari: Matsuura and Sutô 1935
	18				Japan, Hokkaido, Notoro Misaki, Abashiri: Nishikawa 1990
D. julia K.-Pol.	18*				unknown locality: Matveeva and Tikhonova in Bolkhovskikh *et al*. 1969

D. kiusiana Miq.	18	9			Japan, Botanical Garden, Tokyo (cult.): Osawa 1913
D. laureola L.			5.97	2.99	Serbia, Jelasnicka canyon: Siljak-Yakovlev *et al*. 2010
		9			Austria, Vienna: Fuchs 1938
	18				Algeria, Setif region: Ramdani *et al*. 2015
	18				Bulgaria, Forebalkan, Mt Vrachanska: Ivanova and Vladimirov 2007
	18	9			France, Samoëns: Blaise 1959
	18				Greece, Mt. Olympus: Strid and Franzen in Löve 1981
	18				Italy, Sicily, Madonie, Contrada Canale: Lentini *et al*. 1988
	18				Italy, Calabria: Peruzzi and Cesca 2003
	18				Spain, Provincia de Jaén: Löve and Kjellqvist 1974
	18				United Kingdom: Maude 1939
	18				United Kingdom, Dorset, Torbet: Al-Bermani *et al*. 1993
D. longilobata (Lecomte) Turrill	18				unknown locality (cult.): Tan 1980
D. mezereum L.	18				Belarus, Biarezinski Biosphere Reserve: Semerenko 1990, 1993
	18		6.50	3.03	Bosnia and Herzegovina, Bijambare: Siljak-Yakovlev *et al*. 2010
	27		8.80	4.40	Bosnia and Herzegovina, Kladanj: Pustahija *et al*. 2013
	18				Czech Republic, park in Průhonice; N. Moravia, Jeseníky Mts., Malá Morávka: Měsíček 1992
	18		5.51	2.75	Czech Republic, Botanical Garden of the Faculty of Science, Masaryk University, Brno (cult.): Šmarda *et al*. 2019
	18				Finland, Suomusjärvi, Salittu: Sorsa 1962
	18				Greece, Mt. Olympus: Strid and Franzén in Löve 1981
	18	9			Indonesia, Java, Buitenzorg Botanic Gardens (cult.): Strasburger 1909

	18				Poland, Southern Poland, Okocim; Western Carpathians, Babia Góra, Przeginia, Miechów; Wejherowo: Wcisło in Skalińska *et al*. 1976
	18				Slovakia, Arborétum Mlyňany (cult.): Murín and Uhríková in Májovský *et al.* 1970
	18				Slovenia, Zerjav: Druskovic and Lovka in Stace 1995
	18				United Kingdom: Maude 1939, 1940
D. mucronata Royle (syn. *D. angustifolia* C. Koch)		9			Iran, Markazi, Arak, Tureh: Ghaffari 2008
D. odora Thunb.		12,13,14			Japan, Botanical Garden, Tokyo (cult.): Osawa 1913
	28	14			unknown locality: Hiraoka 1958
	27				Japan: Okura and Kono 1959
	28				Japan, Botanical Garden, Tokyo (cult.): Osawa 1913
	27, 28*				unknown locality: Takenaka 1931
	30				unknown locality: Yamaha 1927
D. oleoides Schreb.		9			India, Kashmir, Srinagar Fields: Bhat *et al*. in Löve 1974
	18				Bulgaria, Pirin Mts., Pogledec: Nikolov 1991
	18		10.68	5.34	Lebanon, Quammouah: Bou Dagher-Kharrat *et al*. 2013
D. oleoides subsp. *transcaucasica* (Pobed.) Halda (syn. *D. transcaucasica* (Pobed.))	18				Armenia, Aragac Mt.: Pogosian *et al*. 1974
D. papyracea Wall. ex G. Don (syn. *D. cannabina* Wall.)		9			unknown locality: Venkateswarlu 1946
		18			Nepal, Godawari: Malla *et al*. in Löve 1977
	18				India, Eastern Himalayas, Tonglu: Roy *et al*. 1988
D. pontica L.	18				Bulgaria, Mt Strandzha: Ivanova and Vladimirov 2007
D. pseudomezereum A. Gray	18	9			Japan, Botanical Garden, Tokyo (cult.): Osawa 1913
D. × *reichsteinii* Landolt & E. Hauser	36				Italy, Campione di Garda: Landolt and Hauser 1981
D. rodriguezii Texidor	18				Spain, Balearic islands, Colom Island: Cardona 1977

D. sericea Vahl (syn. *D. collina* J. E. Smith ex Dickson)		9			Greece, Crete: De Montmollin 1984
		9			USA, Harvard, Arnold Arboretum (cult.): Nevling in Löve and Solbrig 1964
D. sericea subsp. *pseudosericea* (Pobed.) Halda (syn. *D. pseudosericea* Pobed.)	18				Georgia, Megrelia: Gagnidze 1983
D. tangutica Maxim (syn. *D. wilsonii* Rehder)	18				unknown locality (cult.): Blaise 1959
D. × *hybrida* Lindl. (*D. odora* Thunb. × *D. sericea* 'Collina' Vahl.)		9			USA, Harvard, Arnold Arboretum (cult.): Nevling in Löve 1966

References

Al-Bermani AKKA, Al-Shammary KIA, Gornall RJ, Bailey JP. 1993. Contribution to a cytological catalogue of the British and Irish flora, 3. *Watsonia* 19: 169–171.

Baksay L. 1956. Cytotaxonomical studies on the flora of Hungary. *Annales Historico-Naturales Musei Nationalis Hungarici* 7: 321–334.

Bhat BK, Bakshi SK, Kaul MK. 1974. Reports. In: Löve Á, ed. IOPB chromosome number reports XLIII. *Taxon* 23:193–196.

Björkqvist I, von Böthmer R, Nilsson Ö, Nordenstam B. 1969. Chromosome numbers in Iberian angiosperms. *Botaniska Notiser* 122:271–283.

Blaise S. 1959. Contribution a l'étude caryologique et palynologique de quelques Thymelaeaceae. *Revue Générale de Botanique* 66:110–161.

Bou Dagher-Kharrat M, Abdel-Samad N, Douaihy B, Bourge M, Fridlender A, Siljak-Yakovlev S, Brown SC. 2013. Nuclear DNA C-values for biodiversity screening: case of the Lebanese flora. *Plant Biosystems* 147:1228–1237.

Cardona MÀ. 1977. A contribution to the cytotaxonomic knowledge of the Balearic flora II. *Col·loquis de la Societat Catalana de Biologia* 10–11:51–67.

De Montmollin B. 1984. Étude cytotaxonomique de la flore de la Crète. II. Nombres chromosomiques. *Botanica Helvetica* 94:261–267.

De Montmollin B. 1986. Étude cytotaxonomique de la flore de la Crète. III. Nombres chromosomiques. *Candollea* 41:431–439.

Delay J. 1969. Determinations nouvelles. *Informations Annuelles de Caryosystématique et Cytogénétique* 3:13–16.

Diosdado JC, Santa-Bárbara C, Vioque J, Juan R, Pastor J. 1993. Números cromosomáticos para la flora Española. 691–719. *Lagascalia* 17:173–184.

Druskovic B, Lovka M. 1995. Reports. In: Stace CA, ed. IOPB chromosome data 9. *International Organization of Plant Biosystematists: Newsletter (Zurich)* 24:15–19.

Fuchs A. 1938. Beiträge zur Embryologie der Thymelaeaceae. *Österreichische Botanische Zeitschrift* 87:1–41.

Gagnidze RI. 1983. Investigatio caryologica complexuum floristicorum calcareorum transcaucasiae occidentalis. *Zametki po Sistematike i Geografii Rastenii Tbilisi* 39:21–38.

Ghaffari SM. 2008. Chromosome records for some plant species from Iran. *The Iranian Journal of Botany* 14:39–46.

Hiraoka T. 1958. Somatic syndesis in *Daphne odora* I. The chromosome behaviour in mitosis. *Proceedings of the Japan Academy* 34:700–705.

Ivanova D, Vladimirov V. 2007. Chromosome numbers of some woody species from the Bulgarian flora. *Phytologia Balcanica* 13:205–207.

Krahulcová A. 1991. Selected chromosome counts of the Czechoslovak flora III. *Folia Geobotanica & Phytotaxonomica* 26:225–368.

Landolt E, Hauser E. 1981. *Daphne reichsteinii* sp. nov., a new hybridogenous *Daphne* species from the region of Lake Garda. *Berichte des Geobotanischen Institutes der Eidg. Techn. Hochschule, Stiftung Rübel* 48:36–47.

Lentini F, Romano S, Raimondo FM. 1988. Numeri cromosomici per la flora italiana: 1185–1196. *Informatore Botanico Italiano* 20:637–646.

Löve Á, Kjellqvist E. 1974. Cytotaxonomy of Spanish plants. IV. Dicotyledons: Caesalpiniaceae – Asteraceae. *Lagascalia* 4:153–211.

Malla SB, Bhattarai S, Gorkhali M, Saiju H, Singh MP. 1977. Reports. In: Löve Á, ed. IOPB Chromosome number reports LVII. Taxon 26:444–446.

Matsuura H, Sutô T. 1935. Contributions to the idiogram study in phanerogamous plants I. *Journal of the Faculty of Science, Hokkaido Imperial University. Ser. 5, Botany* 5: 33–75.

Matveeva TS, Tikhonova AD. 1969. *Daphne julia*. In: Bolkhovskikh ZV, Grif VG, Zakharieva OI, Matveeva TS. 1969. *Chromosome numbers of flowering plants.* Leningrad: Nauka, V. L. Komarov Botanical Institute of the Academy of Sciences of the U.S.S.R., 707.

Maude PF. 1939. The Merton catalogue. a list of the chromosome numerals of species of British flowering plants. *The New Phytologist* 38:1–31.

Maude PF. 1940. Chromosome numbers in some British plants. *The New Phytologist* 39:17–32.

Měsíček J. 1992. *Daphne mezereum* L. In: Měsíček J. and Javůrková-Jarolímová V, eds. *List of chromosome numbers of the Czech vascular plants*. Praha: Academia.

Murín A. 1978. *Daphne arbuscula*. In: Májovský J *et al*. Index of chromosome numbers of Slovakian flora (Part 6). *Acta Faultatis Rerum Naturalium Universitas Comenianae Botanica* 26:1–42.

Murín A. 1990. Karyology of an endemic species *Daphne arbuscula* Čelak. *Acta Faultatis Rerum Naturalium Universitas Comenianae Botanica* 37:35–40.

Murín A, Uhríková A. 1970. *Daphne mezereum* L. In: Májovský J *et al*. Index of chromosome numbers of Slovakian flora (Part 1). *Acta Faultatis Rerum Naturalium Universitas Comenianae Botanica* 16:1–26.

Natarajan G. 1978. Reports. In: Löve Á, ed. IOPB chromosome number reports LXII. *Taxon* 27:526–531.

Nevling LI. 1962. Documented chromosome numbers of plants. *Madroño* 16:266–268.

Nevling LI. 1964. In: Löve Á, Solbrig OT, eds. IOPB chromosome number reports II. *Taxon* 13:201–209.

Nevling LI. 1966. Reports. In: Löve Á, ed. IOPB chromosome number reports VI. *Taxon* 15:117–128.

Nevling LI, Santamour FS, Evers RA. 1962. Chromosome counts of two Thymelaeaceae. *Rhodora* 64:277–283.

Nikolov NA. 1991. Chromosome numbers of Bulgarian angiosperms from North Pirin Mountain: Reserve "Bajuvi Dupki-Dzindzirica". *Fitologija* 41:70–75.

Nishikawa T. 1990. Chromosome counts of flowering plants of Hokkaido (13). *Journal of Hokkaido University of Education, Section II B* 40:19–30.

Okura E, Kono M. 1959. Cytogenetical consideration of *Daphne odora* Thunb. based on its karyotype. *Biological Journal of Okayama University* 5:51–56.

Osawa J. 1913. On the development of the pollen-grain and embryo-sac of *Daphne,* with special reference to the sterility of *Daphne odora*. *Journal of the College of Agriculture, Imperial University of Tokyo* 4:37–264.

Pastor J, Fernández I, Díez M. 1988. Números cromosómicos para la flora Española. 528-543. *Lagascalia* 15:124–129.

Peruzzi L, Cesca G. 2003. Reports 1348-1356. In: Kamari G, Blanch C, Garbari F, eds. Mediterranean chromosome number reports 13. *Flora Mediterranea* 13:373–382.

Pogosian AI, Narinian SG, Voskanian VE. 1974. K kariogeograficheskomu izucheniiu nekotorykh vidov rastenii gory Agarats. *Biologicheskii Zhurnal Armenii* 27:102–104.

Pustahija F, Brown SC, Bogunic F, Bašic N, Muratovic E, Ollier S, Hidalgo O, Bourge M, Stevanovic V, Sijak-Yakovev S. 2013. Small genomes dominate in plants growing on serpentine soils in West Balkans, an exhaustive study of 8 habitats covering 308 taxa. *Plant and Soil* 373:427–453.

Ramdani M, Lograda T, Chalard P, Figueredo G. 2015. Phytochemistry, antibacterial activity and chromosome number of two species of *Daphne* from Algeria. *International Journal of Advances in Pharmacy, Biology and Chemistry* 4:7–17.

Romano S, Mazzola P, Raimondo FM. 1986. Numeri cromosomici per la flora italiana: 1070–1081. *Informatore Botanico Italiano* 18:159–167.

Roy SC, Ghosh S, Chatterjee A. 1988. A cytological survey of eastern Himalayan plants. II. *Cell and chromosome research* 11:93–97.

Semerenko LV. 1990. Chromosome numbers of some flowering plants from the Berezinsky Biosphere Reservation (the Byelorussian Soviet Socialist Republic). *Botanicheskii Zhurnal* 75:279–282.

Semerenko LV. 1993. *Daphne mezereum*. In: Agapova ND, ed. *Numeri Cromosomatum Magnoliophytorum Florae URSS, Moraceae – Zygophyllaceae*. Leningrad: Nauka, 364.

Siljak-Yakovlev S, Pustahija F, Šolic EM, Bogunic F, Muratovic E, Bašic N, Catrice O, Brown SC. 2010. Towards a genome size and chromosome number database of Balkan flora: C-values in 343 taxa with novel values for 242. *Advanced Science Letters* 3:190–213.

Sokolovskaya AP, Strelkova OS. 1940. Karyological investigations of the alpine flora on the main Caucasus range and the problem of geographical distribution of polyploids. *Doklady Akademii Nauk SSSR* 29:415–418.

Sorsa V. 1962. Chromosomenzahlen finnischer Kormophyten. I. *Annales Academiae Scientiarum Fennicae, Series A. 4: Biologica* 58:1–14.

Strasburger E. 1909. Zeitpunkt der Bestimmung des Geschlechts, Apogamie. Parthenogenesis und Reduktionsteilung. *Der Histologischen Beiträge* 7:1–124.

Strid A, Franzén R. 1981. Reports. In: Löve Á, ed. IOPB chromosome number reports LXXIII. *Taxon* 30:829–842.

Šmarda P, Knápek O, Březinová A, Horová L, Grulich V, Danihelka J, Veselý P, Šmerda J, Rotreklová O, Bureš P. 2019. Genome sizes and genomic guanine+cytosine (GC) contents of the Czech vascular flora with new estimates for 1700 species. *Preslia* 91:117–142.

Takenaka Y. 1931. Further reports of the cytological investigations on the sterile plants. *Journal of Chosen Natural History Society*.12:25–41.

Tan K. 1980. Studies in the Thymelaeaceae I: germination, seedlings, fruits and seeds. *Notes from the Royal Botanic Garden, Edinburgh* 38:149–164.

Uhríková A, Májovsky J. 1978. Reports. In: Löve Á, ed. IOPB chromosome number reports LXI. *Taxon* 27:378–379.

Urbani M. 1992. Ricerche biosistematiche e corologiche sulle Thymelaeaceae in Italia:1. *Daphne alpina* L. *Webbia* 46:203–217.

Van Loon JC. 1982. Reports. In: Löve Á, ed. IOPB chromosome number reports LXXVII. *Taxon* 31:763–764.

Venkateswarlu J. 1946. Chromosome numbers of two members of Thymelaeaceae. *Current Science* 25:142.

Wcisło H. 1976. In: Skalińska M, Jankun A, Wcisło H *et al*., eds. Further studies in chromosome numbers of Polish angiosperms. Eleventh contribution. *Acta Biologica Cracoviensia, Series Botanica* 19:107–148.

Wcisło H. 1990. In: Pogan E, Jankun A, Wcisło H *et al*., eds. Further studies in chromosome numbers of Polish angiosperms. Part XXIII. *Acta Biologica Cracoviensia, Series Botanica* 32:171–188.

Yamaha G. 1927. Experimentelle zytologische Beiträge III. Mitteilung. Über die Wirkung einiger Chemikalien auf die Pollenmutterzellen von *Daphne odora* Thunb. *The Botanical Magazine, Tokyo* 41:181–211.
